# Distinct SNP Combinations Confer Susceptibility to Urinary Bladder Cancer in Smokers and Non-Smokers

**DOI:** 10.1371/journal.pone.0051880

**Published:** 2012-12-20

**Authors:** Holger Schwender, Silvia Selinski, Meinolf Blaszkewicz, Rosemarie Marchan, Katja Ickstadt, Klaus Golka, Jan G. Hengstler

**Affiliations:** 1 Mathematical Institute, Heinrich Heine University Düsseldorf, Düsseldorf, Germany; 2 Leibniz Research Centre for Working Environment and Human Factors (IfADo), Dortmund, Germany; 3 Faculty of Statistics, TU Dortmund University, Dortmund, Germany; Johns Hopkins University, United States of America

## Abstract

Recently, genome-wide association studies have identified and validated genetic variations associated with urinary bladder cancer (UBC). However, it is still unknown whether the high-risk alleles of several SNPs interact with one another, leading to an even higher disease risk. Additionally, there is no information available on how the UBC risk due to these SNPs compare to the risk of cigarette smoking and to occupational exposure to urinary bladder carcinogens, and whether the same or different SNP combinations are relevant in smokers and non-smokers. To address these questions, we analyzed the genotypes of six SNPs, previously found to be associated with UBC, together with the *GSTM1* deletion, in 1,595 UBC cases and 1,760 controls, stratified for smoking habits. We identified the strongest interactions of different orders and tested the stability of their effect by bootstrapping. We found that different SNP combinations were relevant in smokers and non-smokers. In smokers, polymorphisms involved in detoxification of cigarette smoke carcinogens were most relevant (*GSTM1*, rs11892031), in contrast to those in non-smokers with *MYC* and *APOBEC3A* near polymorphisms (rs9642880, rs1014971) being the most influential. Stable combinations of up to three high-risk alleles resulted in higher odds ratios (OR) than the individual SNPs, although the interaction effect was less than additive. The highest stable combination effects resulted in an OR of about 2.0, which is still lower than the ORs of cigarette smoking (here, current smokers' OR: 3.28) and comparable to occupational carcinogen exposure risks which, depending on the workplace, show mostly ORs up to 2.0.

## Introduction

Urinary bladder cancer (UBC) is the ninth most common cancer worldwide [1]. The strongest known risk factors include cigarette smoking, occupational exposure to urinary bladder carcinogens, and male gender. It is well established that a deletion variant of the detoxifying phase II metabolizing enzyme glutathione S-transferase M1 (*GSTM1*), in addition to N-acetyltransferase 2 (*NAT2*) slow acetylation are associated with increased urinary bladder cancer risk [2]–[6]. Recently, further genetic variants have been identified and validated in several genome-wide association studies [7]–[12] and were extended to occupational exposure [13]–[15].

The recently discovered SNPs and the corresponding genes have already been comprehensively discussed [1]. Briefly, rs1014971 maps to a non-genic region of chromosome 22q13.1 [9] close to *CBX6* and *APOBEC3A*. Chromobox homolog 7 (*CBX7*) positively regulates E-cadherin expression by interacting with histone deacetylase 2 [16]. This possibly explains why loss of *CBX7* expression is associated with a highly malignant phenotype of carcinomas. Overexpression of *APOBEC3* genes may lead to genetic instability [17]. Rs11892031 is located on chromosome 2q37 in an intronic region of the UDP-glucuronosyltransferase 1A (*UGT1A*) locus. UGT1A is a phase II metabolizing enzyme that catalyzes the glucuronidation and elimination of numerous xenobiotics [18], [19]. Rs1495741 (on chromosome 8p22) is known as a tagging SNP of N-acetyltransferase 2 (*NAT2*) that distinguishes between fast and slow acetylators [20], [21]. Compared to fast acetylators, slow acetylators have an increased bladder cancer risk, probably because of their decreased ability to efficiently detoxify aromatic amines. Rs710521[A] on chromosome 3q28 close to *TP63* is associated with urinary bladder cancer risk [7], [14]. *TP63* shows strong homology to the tumour suppressor P53 [22,23; review: 1]. Rs8102137 on 19q12 maps to Cyclin E (*CCNE1*) which controls cell cycle progression at the G1/S transition [24; review: 1]. Rs9642889, 30 kb upstream of the *MYC* gene on chromosome 8q24.21, confers susceptibility to bladder cancer and influences expression of *MYC* [7], [13]. The well-known proto oncogene *MYC* is involved in the control of proliferation and cell cycle progression [25]. Deletion of the detoxifying phase II enzyme glutathione S-transferase M1 (*GSTM1*) on chromosome 1q13.3 leads to a decreased detoxification of numerous xenobiotics, including polycyclic aromatic hydrocarbons that are known bladder carcinogens [13], [26]. Although the association of each of these SNPs with urinary bladder cancer risk has been validated and confirmed in several independent cohorts, it is still not known if there is an interaction among the high-risk alleles, and if their influence differs between smokers and non-smokers. Therefore, we determined the most influential genetic variants (rs1014971, rs11892031, rs1495741, rs710521, rs8102137, rs9642880, and *GSTM1*) in 1,595 bladder cancer cases and 1,760 controls. We performed interaction analyses addressing the following questions: Are there specific and stable SNP interactions resulting in higher odds ratios than individual SNPs? If so, are these SNP combinations identical or distinct between smokers and non-smokers? Finally, how high is the combined genetic (SNP-based) risk compared to that of cigarette smoking and occupational exposure? We report that specific SNP combinations show a higher UBC risk than individual SNPs, where distinct SNP combinations confer susceptibility in smokers and non-smokers. These risks are, however, still small when compared to that of cigarette smoking.

## Materials and Methods

### Ethics Statement

The sample collection by the Leibniz Research Centre for Working Environment and Human Factors (*IfADo*) was approved by the ethics commission of the Leibniz Research Centre for Working Environment and Human Factors (Ethikkommission des Leibniz-Instituts für Arbeitsforschung an der TU Dortmund) and the institutional review board of the Leibniz Research Centre for Working Environment and Human Factors (Wissenschaftlicher Beirat des Leibniz-Instituts für Arbeitsforschung an der TU Dortmund). All participants provided their written informed consent.

### Patients

To investigate whether there is a combined effect of SNPs associated with UBC, a total of 1,595 UBC cases of European descent and 1,760 controls of European descent from four case-control series collected by the Leibniz Research Centre for Working Environment and Human Factors (*IfADo*) were genotyped at the glutathione S-transferase M1 (*GSTM1*) and six SNPs (rs1014971, rs11892031, rs1495741, rs710521, rs8102137, rs9642880) previously identified in genome-wide association studies to be associated with UBC [7], [9].

This data set comprised confirmed urinary bladder cancer cases and controls without malignant disease from the Department of Urology, Semmelweis University, Budapest, Hungary (“Hungary”; 246 cases and 78 controls), the Department of Urology, Paul Gerhardt Foundation, Lutherstadt Wittenberg, Germany (“East Germany”; 218 cases and 213 controls), the “West Germany – Ongoing” case-control series conducted at five hospitals (in total, 646 cases and 525 controls), and the “West Germany – Industrial” burdened case-control series (in total, 485 cases –111 UBC cases from the Department of Urology, Klinikum Dortmund, Germany, and 374 UBC cases surveyed for recognition of an occupational disease – and 944 controls). Information on profession obtained by questionnaire was available for the “East Germany” case-control series only (information on profession: 216 cases and 211 controls) [27], [28]. Detailed descriptions of these four case-control series can be found in [15].

Patients’ characteristics, such as distribution of gender, age at diagnosis for cases and age at examination for controls, as well as numbers of cases and controls in the individual case-control series, are summarized in Tables S1, S2, and S3. 101 cases and 37 controls with unknown smoking habits were excluded from the interaction analysis in the study groups, leading to a total of 1,494 cases and 1,723 controls that were finally considered to determine the impact of SNP combinations on the UBC risk.

### Polymorphisms

Isolation of genomic DNA of leucocytes was performed according to standard procedures. Genotypes of the SNPs rs1014971, rs11892031, rs1495741, rs710521, rs8102137, and rs9642880 were detected via TaqMan® Assay. Details of the SNPs are given in Appendix S1 and Table S4.

The homozygous *GSTM1* deletion was detected by the amplification of the *GSTM1* DNA sequence segment with 218 base pairs by means of PCR [29], [30]. After gel-electrophoresis using ethidium bromide, the DNA product was detected using UV light. This method helped determine whether at least one copy of the *GSTM1* gene was present or totally missing.

### Statistical Analysis

Cigarette smoking was defined as non-smokers, former smokers, i.e. smokers that quit smoking at least one year before diagnosis (cases) or examination (controls), and current smokers. Former and current smokers were pooled together as “ever smokers”. Analyses were performed stratified for non-smokers, former smokers and current smokers as well as for ever smokers. Analyses on the combined ever smokers groups reflect the past exposure to bladder carcinogens accounting for the latency time of bladder cancer of several decades. Age was defined as “age at diagnosis” for the cases and “age at examination” for the control persons.

Deviations from Hardy-Weinberg equilibrium (HWE) were checked in each study group and separately for cases and controls using χ^2^ tests (for the results, see Table S5). Associations of polymorphisms and smoking habits with UBC were evaluated applying χ^2^ tests, odds ratios (OR), and 95% confidence intervals (95% CI). Moreover, ORs and 95% CIs adjusted for age, gender, smoking habits, and study site were estimated using logistic regression.

The ORs of the individual polymorphisms, and combinations of these polymorphisms in the total cohort as well as in subgroups defined by the smoking status of the subjects, were determined by considering the dominant and recessive effects of the SNPs. For each interaction of *p* polymorphisms (*p* = 2, …, 7), the ten combinations showing the OR with the lowest p-values were identified in each of the subgroups. To check whether it is appropriate to compute p-values for higher-order SNP interactions based on a χ^2^ distribution with one degree of freedom, we also determined permutation p-values and compared these with the parametric p-values. Additionally, a bootstrap strategy was used to investigate the stability of the ORs of the SNP combinations of different sizes in the subgroups. To achieve this, 500 bootstrap samples were drawn from the respective subgroup and counted to determine how often the top 10 SNP combinations from the original analysis appeared among the top 10, top 20, and top 50 SNP combinations (of the same number of SNPs) from the analyses of the corresponding 500 bootstrap samples.

To test whether the OR of a certain SNP combination differs between the ever smokers and the non-smokers, logistic regression models were fitted containing parameters for the respective SNP combination, smoking status, and the interaction between these two factors. The standard test for the interaction parameter in this logistic regression model was used to test whether the ORs differ significantly between smokers and non-smokers. Details on this and other statistical analyses can be found in Appendix S2.

Population attributable risks (PAR) indicating the proportion of cases that could be attributed to a certain risk factor, and combined PARs for two or more independent risk factors were calculated according to [31]. The PARs of the individual polymorphism were calculated based on adjusted and unadjusted ORs. Combined PARs were determined based on the adjusted ORs of the homozygous and heterozygous vs. the reference genotypes of each SNP. ORs were adjusted for age, gender, smoking habits, study site (in case of combined study groups) and all measured polymorphisms but rs11892031, as this SNP has a rather protective effect in about 16% of the population of European descent [32]. All four study groups were used to determine the PAR due to smoking habits and genetic risk factors in the present study, whereas the PAR for certain professions was based on the “East Germany” case-control series only.

For an overview of UBC risk factors from the literature, we performed an extensive literature search using PubMed. We included the relevant papers on UBC causes in populations of European descent. If possible, we used the given adjusted ORs to determine the PAR from published studies. Otherwise, unadjusted ORs or ORs calculated from the published frequencies were used. Estimation of ORs of combined genetic risk factors was done for varying frequencies assuming a PAR of 30%.

## Results

### Analysis of ORs of SNP Combinations

Currently, it is unknown whether genetic variants associated with increased UBC risk interact with one another resulting in higher odds ratios (OR) for combinations than for individual SNPs. Therefore, we analyzed the ORs from combinations of up to seven polymorphisms that were previously found to be individually associated with UBC [2], [7], [9], [20]. The ORs as well as the corresponding 95% confidence intervals (95% CI) and p-values for the individual SNPs, determined in the analysis of our total study group and subgroups defined by the smoking habits, are summarized in Table S6.

**Figure 1:**
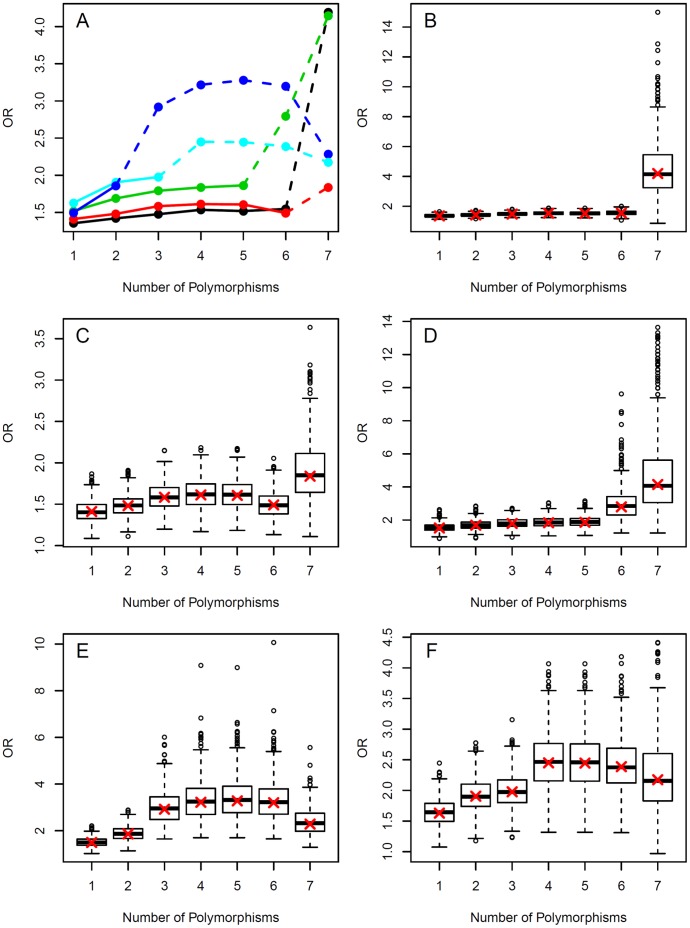
Optimal odds ratios for combinations of one to seven polymorphisms. For the computation of the optimal odds ratios (OR), all possible combinations of one to seven of the polymorphisms rs1014971, rs9642880, rs710521, rs8102137, rs11892031, rs1495741 and *GSTM1* were considered. (A) Profile plots for the odds ratios in the total group (black line) and the subgroups of ever smokers (red line), current smokers (green), former smokers (blue) and non-smokers (cyan). The lines were included for clarity of information and not to suggest a continuous development. Dashed lines indicate when number of cases and/or number of controls fall below 100. In these situations, the corresponding odds ratios should be interpreted with caution. (B)–(F): For the optimal combinations shown in (A), box plots of odds ratios computed in 500 bootstrap samples from (B) the total group, (C) the ever smokers, (D) the current smokers, (E) the former smokers and (F) the non-smokers. In twelve of the bootstrap samples (all but one in the analyses of the seven-way interactions in the total and the smoker group), the odds ratios were larger than 15. For a better presentation, these odds ratios are not displayed in the corresponding box plots. The crosses mark the odds ratios of the optimal combinations in the original analysis. The corresponding plots of the test statistics are shown in Figure S1.

Analyzing the SNP combinations, the ORs of the optimal SNP combinations, in general, increased with the numbers of combined SNPs (Figure 1A). However, case numbers of the high-risk alleles decreased rapidly when several SNPs were combined, thus leading to relatively high variability of the odds ratios in the bootstrap sample (Figure 1B–F). Here the variation typically increased with decreasing number of subjects. In contrast to the ORs, the Wald statistics corresponding to the ORs increased from individual SNPs to combinations of three polymorphisms. However, no further increase was observed (Figure S1), which is again due to high variances and small sample sizes.

In Tables 1, 2, 3 and 4, the ORs with 95% CIs and the p-values of the ten combinations of two and three polymorphisms with the smallest p-values found in the analysis of the ever smokers and the non-smokers are shown. The ORs of the top ten individual effects as well as the top ten two-way and three-way interactions in the total group and in the smoker subgroups are presented in Tables S7, S8, S9, S10, S11, S12, S13, S14, S15, S16, S17, S18, S19 S20 and S21. Additionally, we summarized how often the seven polymorphisms occur in the top ten two-way and three-way interactions in the different subgroups (Table 5).

**Table 1:** Top ten two-way interactions found in the analysis of the ever smokers.

SNP combination	OR (95% CI)	P-value
rs11892031 [A/A] × *GSTM1* null	1.48 (1.25–1.76)	0.0024
rs8102137 [C/T, T/T] × *GSTM1* null	1.51 (1.25–1.82)	0.0040
rs710521 [A/A, A/G] × *GSTM1* null	1.46 (1.22–1.73)	0.0062
rs710521 [A/A, A/G] × *GSTM1* present	0.69 (0.58–0.83)	0.0105
rs9642880 [G/G, G/T] × *GSTM1* present	0.69 (0.57–0.82)	0.0113
rs11892031 [A/A, A/C] × *GSTM1* present	0.70 (0.59–0.84)	0.0185
rs11892031 [A/A, A/C] × *GSTM1* null	1.42 (1.19–1.69)	0.0204
rs1014971 [C/C, C/T] × *GSTM1* present	0.71 (0.60–0.84)	0.0303
rs1495741 [A/A, A/G] × *GSTM1* null	1.40 (1.18–1.66)	0.0398
rs1014971 [C/C, C/T] × *GSTM1* null	1.38 (1.16–1.64)	0.0703

The top ten of the 288 possible two-way interactions comprised of the six SNPs and *GSTM1* as well as their odds ratios (OR) with 95% confidence intervals (CI) are listed in order of their p-values, where the p-values were adjusted for multiple comparisons by the Bonferroni correction.

**Table 2:** Top ten two-way interactions found in the analysis of the non-smokers.

SNP combination	OR (95% CI)	P-value
rs9642880 [G/T, T/T] × rs1014971 [C/C]	1.91 (1.44–2.51)	0.0015
rs9642880 [G/G, G/T] × rs1014971 [C/T, T/T]	0.56 (0.43–0.74)	0.0112
rs710521 [A/A, A/G] × rs1014971 [C/C]	1.68 (1.28–2.20)	0.0458
rs1014971 [C/C] × rs1495741 [A/A, A/G]	1.66 (1.27–2.16)	0.0524
rs1014971 [C/C] × rs11892031 [A/A, A/C]	1.65 (1.27–2.16)	0.0564
rs1014971 [C/T, T/T] × rs8102137 [C/C, C/T]	0.61 (0.46–0.79)	0.0640
rs1014971 [C/C] × rs11892031 [A/A]	1.65 (1.26–2.15)	0.0761
rs9642880 [T/T] × rs710521 [A/A, A/G]	1.75 (1.29–2.37)	0.0827
rs1014971 [C/T, T/T] × rs1495741 [A/A, A/G]	0.62 (0.47–0.81)	0.1051
rs1014971 [C/C] × *GSTM1* null	1.73 (1.28–2.35)	0.1111

The top ten of the 288 possible two-way interactions comprised of the six SNPs and *GSTM1* as well as their odds ratios (OR) with 95% confidence intervals (CI) are listed in order of their p-values, where the p-values were adjusted for multiple comparisons by the Bonferroni correction.

**Table 3:** Top ten three-way interactions found in the analysis of the ever smokers.

SNP combination	OR (95% CI)	P-value
rs8102137 [C/T, T/T] × rs11892031 [A/A] × *GSTM1* null	1.58 (1.30–1.92)	0.0059
rs710521 [A/A, A/G] × rs11892031 [A/A] × *GSTM1* null	1.51 (1.26–1.80)	0.0080
rs710521 [A/A, A/G] × rs8102137 [C/T, T/T] × *GSTM1* null	1.55 (1.28–1.88)	0.0135
rs9642880 [G/G, G/T] × rs710521 [A/A, A/G] × *GSTM1* present	0.66 (0.55–0.79)	0.0171
rs8102137 [C/T, T/T] × rs1495741 [A/A, A/G] × *GSTM1* null	1.52 (1.26–1.84)	0.0248
rs8102137 [C/T, T/T] × rs11892031 [A/A, A/C] × *GSTM1* null	1.50 (1.25–1.81)	0.0315
rs1014971 [C/C, C/T] × rs11892031 [A/A] × *GSTM1* null	1.46 (1.22–1.74)	0.0419
rs9642880 [G/G, G/T] × rs11892031 [A/A, A/C] × *GSTM1* present	0.68 (0.57–0.82)	0.0520
rs710521 [A/A, A/G] × rs11892031 [A/A, A/C] × *GSTM1* null	1.45 (1.22–1.72)	0.0552
rs710521 [A/A, A/G] × rs1014971 [C/C, C/T] × *GSTM1* present	0.69 (0.57–0.82)	0.0582

The top ten of the 1,760 possible three-way interactions comprised of the six SNPs and *GSTM1*, as well as their odds ratios (OR) with 95% confidence intervals (CI) are listed in order of their p-values, where the p-values were adjusted for multiple comparisons by the Bonferroni correction.

**Table 4:** Top ten three-way interactions found in the analysis of the non-smokers.

SNP combination	OR (95% CI)	P-value
rs9642880 [G/T, T/T] × rs710521 [A/A, A/G] × rs1014971 [C/C]	1.98 (1.49–2.63)	0.0044
rs9642880 [G/T, T/T] × rs1014971 [C/C] × rs1495741 [A/A, A/G]	1.95 (1.47–2.58)	0.0054
rs9642880 [G/T, T/T] × rs1014971 [C/C] × rs11892031 [A/A, A/C]	1.93 (1.46–2.55)	0.0061
rs9642880 [G/T, T/T] × rs1014971 [C/C] × *GSTM1* null	2.21 (1.58–3.10)	0.0070
rs9642880 [G/G, G/T] × rs1014971 [C/T, T/T] × rs8102137 [C/C, C/T]	0.54 (0.40–0.71)	0.0318
rs9642880 [G/G, G/T] × rs710521 [A/A, A/G] × rs1014971 [C/T, T/T]	0.54 (0.40–0.71)	0.0325
rs9642880 [G/G, G/T] × rs1014971 [C/T, T/T] × rs1495741 [A/A, A/G]	0.56 (0.42–0.74)	0.0735
rs710521 [A/A, A/G] × rs1014971 [C/C] × *GSTM1* null	1.93 (1.41–2.64)	0.0773
rs9642880 [G/T, T/T] × rs1014971 [C/C] × rs11892031 [A/A]	1.80 (1.35–2.40)	0.0954
rs710521 [A/A, A/G] × rs1014971 [C/C] × rs11892031 [A/A]	1.74 (1.33–2.29)	0.1142

The top ten of the 1,760 possible three-way interactions comprised of the six SNPs and *GSTM1*, as well as their odds ratios (OR) with 95% confidence intervals (CI) are listed in order of their p-values, where the p-values were adjusted for multiple comparisons by the Bonferroni correction.

**Table 5:** Number of times the considered polymorphisms appear in the ten top two- and three-way interactions when analyzing the different smoker groups.

Polymorphism	Total	Ever	Current	Former	Never
*GSTM1*	10 (9)	10 (10)	10 (10)	7 (4)	2 (1)
rs11892031	6 (3)	6 (3)	4 (3)	3 (2)	3 (2)
rs710521	5 (4)	5 (2)	4 (2)	5 (3)	4 (2)
rs9642880	5 (3)	2 (1)	3 (1)	8 (7)	8 (3)
rs8102137	3 (1)	4 (1)	2 (1)	5 (2)	1 (1)
rs1495741	1 (0)	1 (1)	0 (0)	2 (1)	2 (2)
rs1014971	0 (0)	2 (2)	7 (3)	0 (1)	10 (9)

Numbers in brackets are from the analysis of the two-way interactions. Numbers outside the brackets are from the analysis of the three-way interactions. The corresponding groupwise top ten two-way interactions are listed in Tables S12, S13, S14, S15 and S16, and the top ten three-way interactions are presented in Tables S17, S18, S19, S20 and S21.

### Appropriateness of Parametric p-values

Since the p-values were determined using a χ^2^ distribution with one degree of freedom, we examined the suitability of employing such parametric p-values for combinations of several SNPs by comparing these p-values with the corresponding permutation-based p-values. In addition, we computed both the mean and the variance of the test statistics determined in the 100,000 permutations used in the derivation of the latter p-values. The results of these computations are displayed in the supporting information. Figures S2, S3 and S4 indicate that the χ^2^ approximation worked well for most of the combinations of two or three SNPs, and in particular, for the respective top ten combinations. However, the χ^2^ approximation became worse as the number of SNPs forming an interaction increased. Surprisingly, the most extreme differences in p-values for the combinations of two SNPs were larger than the ones for, for example, three-way interactions. This, however, was only relevant for a few combinations.

### Stability of the Estimated ORs

The above results, together with the relatively small case numbers in the subgroups of current, former and non-smoker for combinations of more than three SNPs, led us to focus on the interaction of two and three polymorphisms when we analyzed the stability of the ranks of the SNP combinations in the bootstrap samples (Tables S12, S13, S14, S15, S16 and Tables S17, S18, S19, S20 and S21, respectively). The ranks were very stable considering the individual variables coding for the polymorphisms (Tables S7, S8, S9, S10 and S11). In addition, the top two-way interactions occurred among the top ten interactions in a large majority of the bootstrap samples (Tables S12, S13, S14, S15 and S16). However, the instability of the ranks increased with the number of polymorphisms forming a combination (for example, the ranks for three-way SNP combinations in Tables S17, S18, S19, S20 and S21).

#### Differences in relevant SNP interactions between smokers and non-smokers

Interestingly, different SNP combinations were obtained for non-smokers and smokers. The optimal three-way SNP combinations (resulting in maximal odds ratios) for non-smokers consisted of (i) rs1014971, (ii) rs9642880, and (iii) one of the three SNPs: rs11892031, rs1495741, or rs710521 (Tables 2 and 4 as well as Table 5). In contrast, the optimal combinations for the current smokers were composed of *GSTM1*, rs1014971, and one of the three SNPs: rs11892031, rs710521, and rs9642880 (Table 5 as well as Tables S14 and S19). A similar result was obtained for the ever smokers in which, however, rs1014971 was only rarely present in the top SNP combinations (Table 5 as well as Tables 1 and 3). This SNP also did not appear in any of the top ten three-way interactions in the former smokers (Table 4 as well as Tables S15 and S20). Interestingly, the former smokers showed a mixed SNP pattern of smokers and non-smokers, including *GSTM1* (the top “smoker SNP”), rs9642880 (the second-best scoring “non-smoker SNP”), rs710521 (present in both the smoker and non-smoker SNP combination), as well as rs8102137 (the least or second least important SNP when considering the three-way interactions in non-smokers and current smokers, respectively).

### Comparison with Published Results

Considering the genetic risks due to single well-known and novel polymorphisms, ORs range between null and 1.34 in the present study in accordance with the published results from case-controls studies, meta-analyses and GWAS that did not exceed 1.81 (Table 6). Particularly, UBC risks attributed to *GSTM1* and *NAT2* show a remarkable variation in the literature ranging from 1.28 to 1.70 in case of *GSTM1,* and no considerable effect to mild risks of 1.43 due to slow *NAT2* genotypes not stratified by smoking habits. In terms of relevance for the populations – depending on relative risks and frequency of the risk factors – a considerable fraction of the UBC cases can be attributed to overall genetic risks (30%) or single polymorphisms, in particular *GSTM1* with population attributable risks (PAR) ranging from 13% to 26% (Table 6).

**Table 6:** Population attributable risks and odds ratios due to genetic factors.

	Present study		Published	
Genetic Factors	PAR	OR	PAR	OR
All	–	–	30% [47], [51]	1.04–1.81 [1], [9], [31], [48]
*GSTM1*	13%^a^	1.28	14–26% [47], [48], [54], [55]	1.28–1.70 [47], [48], [54], [55]
*NAT2*	1%^b^	1.02^b^	8.2%^c^ [54]	1.04–1.43 [47], [48], [54], [56]
“wimp” SNPs	33%^d^	1.02–1.34^e^	–	1.11–1.81 [1], [9], [32]
Top 3-way interaction	16%	1.48	–	–

Population attributable risks (PARs) and odds ratios (ORs) were calculated from the data of the present study and summarized from previously published studies for different genetic factors. Numbers in brackets refer to the publications in which the PARs and ORs were published.

a Adjusted for age, gender, smoking habits, all measured SNPs and study site; crude PAR/OR: 16%/1.39; adjusted for age and gender: 16%/1.37; adjusted for all measured SNPs: 15%/1.36.

b Adjusted for age, gender, smoking habits, all measured SNPs and study site; crude PAR/OR: 5%/1.09; adjusted for age and gender: 3%/1.05; adjusted for all measured SNPs: 5%/1.10.

c Data from Moore et al. [48] and Garcia-Closas et al.[47] result in PARs of 2–18%.

d Combined PAR, individual SNP OR and PAR adjusted for age, gender, smoking habits and all measured SNPs.

e Range of individual SNP OR adjusted for age, gender, smoking habits, all measured SNPs and study site depending on the mode of inheritance.

### Comparison of Interaction Effects with Occupational and Environmental Risk

The situation is less clear for risks due to occupational exposure to bladder carcinogens. The risk depends strongly on the population under investigation and time of recruitment, both of which reflects the structure of the local industry and changes in exposure (Table 7). Estimates of overall PARs range from 2–5% for women and 7–10% for men [33], [34] to 20–26% [35]–[37] for highly industrialized areas. Strongly increased risks due to exposure to bladder carcinogens, in particular β-naphthylamine, 4-aminobiphenyl and 4-chloro-o-toluidine, can be found in old studies on highly exposed workers whereas clearly and moderately increased risks are still present but do not exceed ORs of two [38]. Determination of PARs for single professions is hampered by their different frequencies in different regions, though common occupations as painters or hairdressers contribute to 0.2–0.9% of the UBC cases.

**Table 7:** Population attributable risks and odds ratios for different occupational exposures.

		Present study		Published	
Increased risk	Occupation/Exposure	PAR	OR	PAR	OR
	All	–	–	20–26% [35]–[37]	–
				M: 7–10% [33], [34]	
				F: 2–5% [33], [34]	
Moderately	Painter	0.89%	1.38	0.7% [33], [34]	1.17–1.98 [36], [38], [57]–[59] a
	Hairdresser	–	–	0.2% [33], [34]	1.23–2.10 [36], [38], [63]
	Coal miner	2.81%	1.47	–	1.31–2.40 [38], [58], [64], [65]
Clearly	Aluminium Worker^b^	–	–	–	1.50–2.34 [36], [66]
	Rubber Industry	2.80%	1.76	–	1.29–1.30 [36], [38]
	Roofer and Slater	–	–	–	1.70 [36]
Strongly	Benzidine/β-Naphthylamine	–	–	–	1.60 [37]
	Benzidine^c^	–	–	–	30–75 [67], [68]
	β-Naphthylamine^c^	–	–	–	5–200 [68]
	4-Aminobiphenyl^c^	–	–	–	11%^d^ [68]
	4-Chloro-o-toluidine	–	–	–	38–90 [68]

Population attributable risks (PARs) and odds ratios (ORs) were calculated from the data of the present study and summarized from previously published studies for different occupations and occupational exposures, partly stratified by gender (M: Male, F: Female). Numbers in brackets refer to the publications in which the PARs and ORs were published.

a Painters before 1960 had a clearly increased risk: OR = 2.42–2.78 [60]–[62].

b More exactly, Aluminium Workers (Soderberg Processing).

c Results from historical studies.

d Prevalence in exposed workers.

Most UBC cases can clearly be attributed to cigarette smoking (Table 8; present study PAR: 46%; other studies PAR: 50–56%). While current smokers have an approximately 3-fold risk (present study OR = 3.28, other studies OR = 2.77–4.95) of developing UBC – increasing with amount and time – the UBC risk of former smokers decreases to an OR of about two (present study OR = 2.12, other studies OR = 1.74–2.34). Both subgroups contribute almost equally to the UBC cases in the present study (former smokers PAR = 29%, current smokers PAR = 30%), whereas in published studies estimates of the PAR range from 28–40% for former smokers to 39% in current smokers. Interestingly, among men more UBC cases are attributable to smoking (former 41%, current 55%, ever 66%) than among women (former 17%, current 32%, ever 30%).

**Table 8:** Population attributable risks and odds ratios in the different smoker groups.

	Present study		Published	
Smokinghabits	PAR	OR	PAR	OR
Former smokers	30%^a^	2.12^a^	28–40% [48], [53]	1.74–2.34 [48], [53], [71]
			M: 41% [69]	M: 2.74 [69]
			F: 17% [70]	F: 1.42 [70]
Current smokers	29%^b^	3.28^b^	39% [48], [53]	2.77–4.95 [48], [53], [71]
			M: 55% [69]	M: 4.72 [69]
			F: 32% [70]	F: 1.89 [70]
Ever smokers	46%^c^	2.47^c^	50–56% [48], [52], [53]	2.61–2.89 [48], [53]
			M: 66% [69]	M: 3.65 [69]
			F: 30% [70]	F: 1.69 [70]

Population attributable risks (PARs) and odds ratios (ORs) were calculated from the data of the present study and summarized from previously published studies for the different smoker groups, partly stratified by gender (M: Male, F: Female), where non-smokers were used as a reference group having no additional risk. Numbers in brackets refer to the publications in which the PARs and ORs were published.

a Adjusted for age and gender; crude PAR/OR: 39%/2.65; adjusted for age, gender, SNPs: 30%/2.15.

b Adjusted for age and gender; crude PAR/OR: 29%/3.21; adjusted for age, gender, SNPs: 28%/3.17.

c Adjusted for age and gender; crude PAR/OR: 51%/2.83; adjusted for age, gender, SNPs: 46%/2.47.

## Discussion

### Comparison of the Results from Analyzing Non-smokers and Smokers

The distinct SNP patterns for smokers and non-smokers found in our analysis are remarkable, since the genes closest to the top scoring “smoker variants” are involved in the detoxification of carcinogens in cigarette smoke, whereas the top scoring “non-smoker SNPs” are associated with cell cycle control and DNA stability. The deletion variant of *GSTM1*, the polymorphism found in our analysis to be the most important in smokers, results in loss of activity of the phase II metabolizing enzyme glutathione S-transferase M1, which is involved in detoxification of numerous polycyclic aromatic hydrocarbons [39], [40]. The second scoring “smoker variant” rs11892031 is located closest to the *UGT1A* cluster [9]. UDP-glucuronosyltransferase is also a phase II metabolizing enzyme responsible for the conjugation and detoxification of several urinary bladder carcinogens present in cigarette smoke [27], [41]–[45].

In contrast, the two top scoring “non-smoker SNPs” are not involved in carcinogen detoxification. Rs1014971 is located approximately 25 kb centromeric of *APOBEC3A*, which deaminates cytosine to uracil, thereby playing a role in endogenous mutagenesis [1], [9]. The second, rs9642880 is known to influence the expression of the proto oncogene *MYC*, which controls transcription of numerous genes involved in proliferation [7], [13]. This scenario suggests that control factors of proliferation and DNA integrity are critical for susceptibility to bladder cancer in non-smokers. In contrast, enzymes detoxifying cigarette smoke carcinogens seem to be of highest relevance in smokers.

Another striking observation is that the three SNPs forming the optimal three-way SNP combination in non-smokers, i.e. rs9642880[G/T, T/T] x rs710521[A/A, A/G] x rs1014971[C/C], differ from the three polymorphisms composing the optimal three-way interaction in ever smokers, i.e. rs8102137[C/T, T/T] x rs11892031[A/A] x *GSTM1* null. Moreover, the optimal three-SNP combination in non-smokers results in an OR of 1.98 (95% Cl: 1.49–2.63) that is significantly higher (p-value: 1.78×10^−4^) than the OR of this combination in the ever smokers (OR: 1.03, 95% CI: 0.86–1.24). Conversely, the optimal three-SNP combination in ever smokers exhibits an OR of 1.58 (95% Cl: 1.30–1.92), which is substantially, but not significantly (p-value: 0.143) higher than the OR of this three-SNP combination in non-smokers (OR: 1.21; 95% CI: 0.90–1.64). However, cigarette smoking is already associated with an OR of 3.28 (95% Cl: 2.67–4.03) when current smokers are compared to non-smokers in our study population (Table 9). This high OR suggests that under conditions of continuous exposure to cigarette smoke carcinogens, the contribution of the “non-smoker SNPs” with their relatively small influence on cell cycle and DNA integrity control, is of minor relevance.

**Table 9:** Distribution of smoking habits and UBC risk in the present case-control study.

Smoking Habit (*n*_Ca_/*n*_Co_)	Cases	Controls	OR (95% CI)	OR adj (95% CI adj)
Non-smokers (321/752)	21%	44%	(reference)	(reference)
Former smokers (742/656)	50%	38%	2.65 (2.24–3.31)	2.12 (1.78–2.53)
Current smokers (431/315)	29%	18%	3.21 (2.64–3.90)	3.28 (2.67–4.03)
Ever smokers (1173/971)	79%	56%	2.83 (2.42–3.31)	2.47 (2.10–2.90)

For each of the smoker subgroups containing *n_Ca_* cases and *n_Co_* controls, the odds ratios (OR) and their corresponding 95% confidence intervals (95% CI) were computed, both not adjusted and adjusted for age and gender. The latter odds ratios are abbreviated by OR adj.

### Comparison with Published Results

To study the consistency of this observation, we re-visited the data of the genome-wide association study on UBC of Rothman et al. [9] who validated rs9642880 and rs710521 in 3,532 UBC cases and 5,120 controls, and confirmed the impact of the *GSTM1* deletion in 2,480 cases and 3,222 controls. Assuming a multiplicative model, they also obtained higher ORs for non-smokers compared to ever smokers for rs9642880 (1.24 for non-smokers versus 1.16 for smokers) and rs11892031 (1.49 versus 1.31). The higher OR for rs9642880 contradicts the study of Kiemeney et al. [7] who reported no association of rs9642880 with smoking habits. Also, the findings of a higher OR for rs11892031 in non-smokers is in contrast to Tang et al. [32] who found a higher risk in ever smokers (OR = 1.28) than in non-smokers (OR = 1.23) based on a subset of study groups from Rothman et al. (GWAS stage 1 [9]). However, no difference was found for rs710521 (1.13 vs. 1.14) in accordance with the discovery GWAS [7], and an opposite trend was shown for rs1014971 (1.11 vs. 1.16) and the *NAT2* tagging SNP rs1495741 (1.00 vs. 1.18) in accordance with the assumed higher risk of slow acetylators in smokers. Therefore, the difference should still be interpreted with caution until independent confirmatory data are available.

The association among the *GSTM1* null genotype, smoking habits and bladder cancer has been controversial since the first study by Bell et al. in 1993 [2]. In their study, smokers had an OR of 1.8 and non-smokers an OR of 1.3, indicating higher risks in smokers due to the lack of *GSTM1.* However, recent meta analyses and large or pooled studies found no or only weak evidence for an association between *GSTM1* and smoking habits [9], [46]–[48], whereas Rothman et al. [9] reported an even higher OR for non-smokers than for ever smokers (1.71 vs. 1.47). In this context, it should be mentioned that our study groups present a higher proportion of occupationally exposed bladder cancer cases. This may be particularly important for *GSTM1*. For example, it was shown that bladder cancer patients with occupational histories in coal, iron, and steel industries, i.e. exposure to polycyclic aromatic hydrocarbons, presented with high percentages of *GSTM1* null genotypes [49]. Decades after the closure of these industries, the *GSTM1* genotypes were equal in both cases and controls (*GSTM1* null: 52%) [50].

### Gain of Considering SNP Interactions

We have shown that SNP combinations result in less than additive ORs compared to the influence of the individual SNPs. For example, the ORs of the “non-smoker SNPs” rs1014971 and rs9642880 are 1.63 = 1/0.61 and 1.48, respectively, in non-smokers (for all ORs of individual SNPs, see Table S6). In comparison, the combination of both SNPs results in an OR of 1.91 in this subgroup (Table S16), which is larger than the individual effects, but smaller than 1.63+1.48 = 3.11. Adding a third SNP to the rs1014971 × rs9642880 combination results in an increase of only 0.07 (Table S21). The less than additive effect is not surprising considering the relatively high frequencies of the high-risk alleles (rs1014971 [C/C]: 40%; rs9642880 [G/T, T/T]: 71%; rs710521 [A/A, A/G]: 93%) in non-smoking controls and their overlap between individual SNPs (two-way interaction: 27%; three-way interaction 24%). Therefore, it seems unlikely that the addition of further “low impact” or “wimp SNPs” [1] would lead to a relevant increase in the combined ORs in populations of European descent.

### Analysis of Population Attributable Risks

**Figure 2:**
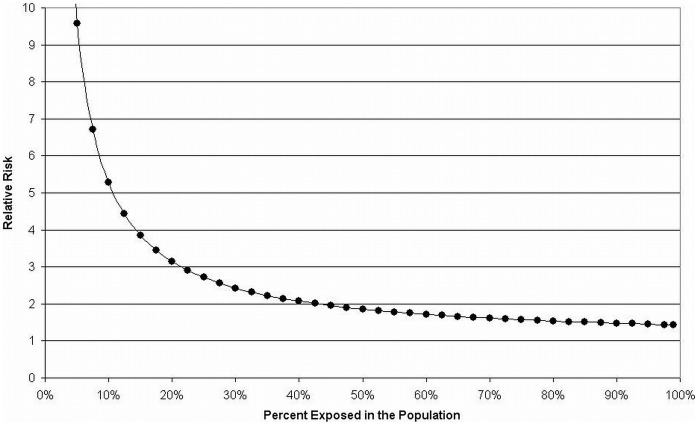
Relative risks and frequency of risk factors assuming a PAR of 30%. Relative risks are calculated depending on the frequency of the risk factor in the population assuming a population attributable risk (PAR) of 30%, corresponding to the supposed PAR of genetic risk factors for UBC. Given a PAR of 30%, the relative risk does not fall below 1.43 if the frequency of the risk factor is present in almost the entire population.

Altogether, it is estimated that up to 30% of bladder cancer cases can be explained by genetic risk factors [47], [51] (see also Table 6), whereas about half of all UBC cases are caused by cigarette smoking [48], [52], [53] (see also Table 8). Estimates of the population attributable risk (PAR) for occupations vary widely, ranging from 7.1% in men and 1.9% in women [34] to 20–26% in both genders [35]–[37] (see also Table 7). The PAR – as a measure of the proportion of cases that could be explained by a certain risk factor – depends on and increases with both the frequency of the risk factor in the population and the relative risk (which is often approximated by the OR). Thus, assuming that the PAR of the genetic risk factors is limited to about 30% in the general population, the OR of the frequent combinations of these polymorphisms must be limited to modest ORs of about two (Figure 2). For instance, a PAR of 30% results from a risk factor present in 40% of the population and a relative risk of 2.1, whereas a risk factor present in 10% of the population requires a relative risk of 5.3 to obtain the same PAR. However, in subgroups different impacts of the genetic risk factors can be observed, not only in terms of relevance of single SNPs and their combinations, but also with respect to their combined attributable risks. In our study, combined PARs for the “wimp SNPs” range from 28% in ever smokers to 43% in non-smokers and also reflect the different impact of genetic risk factors in subpopulations with higher or lower exposure to bladder carcinogens from tobacco smoke.

### Conclusion

In conclusion, we have shown that different types of genetic variants confer different susceptibility to smokers and non-smokers. In addition, the present work fuels the debate regarding the degree to which genetic disposition or environmental exposure contributes to carcinogenesis. Whereas the odds ratio of cigarette smoking is approximately 3.5 for current smokers in most studies, the combined high-risk alleles of the SNPs recently discovered in genome-wide association studies add up to ORs of approximately 2.0. Therefore, the environmental factors seem to have a higher impact on the UBC risk than genetic disposition based on the SNPs derived from recent genome-wide association studies.

## Supporting Information

Figure S1 **Test statistics for the optimal combinations consisting of one to seven SNPs in the bootstrap samples.** Data are shown for (A) the total group, (B) the ever smokers, (C) the current smokers, (D) the former smokers, and (E) the non-smokers. For each of the optimal combinations from in Figure 1A, box plots of the test statistics in the 500 bootstrap samples drawn from the respective subgroups are displayed. The plots correspond to the odds ratios shown in Figure 1B–F. The crosses mark the test statistics for the respective optimal combinations.
(TIFF)

Figure S2 **Mean test statistic over 100,000 permutations of the case-control status.** For the top 100 SNP combinations of each size and in each subgroup, the means over the Wald statistics in 100,000 permutations of the case-control status were computed. The subgroup-wise distributions of these means are shown as box plots, and the subgroup-wise means of the top 10 combinations are marked by red crosses. For a better representation, six outliers (with means smaller than 0.9) were removed from the box plots for the two-way interactions. For reference, the minimum and maximum of the sample means from 100 samples consisting of 100,000 random draws from a χ^2^-distribution with 1 degree of freedom are marked by dashed blue lines. If the χ^2^-approximation is reasonable, the mean test statistic over the 100,000 permutations should be approximately 1, i.e. close to the solid blue lines marking the mean of the χ^2^-distribution with 1 degree of freedom.
(TIFF)

Figure S3 **Variance of the test statistic over 100,000 permutations of the case-control status.** In addition to the mean test statistic displayed in Figure S2, the variances of the test statistics for the top 100 interactions in the different subgroups were computed. The subgroup-wise distributions of these variances are shown as box plots, and the variances of the top ten combinations in the subgroups are marked by red crosses. For a better representation, six outliers (with variances smaller than 1.3) were removed from the box plots for the two-way interactions. For reference, the minimum and maximum of the sample variances from 100 samples consisting of 100,000 random draws from a χ^2^-distribution with 1 degree of freedom are marked by dashed blue lines. If the χ^2^-approximation is reasonable, the variance of the test statistic over the 100,000 permutations should be approximately 2, i.e. close to the solid blue lines marking the variance of the χ^2^-distribution with 1 degree of freedom.
(TIFF)

Figure S4 **Differences between parametric and permutation-based p-values.** Box plots of the differences between the parametric p-values of the top 100 SNP combinations of each size from the analysis of each subgroup and the corresponding p-values computed based on 100,000 permutations of the case-control status. The differences of the respective top ten SNPs are additionally marked by red crosses. Ideally, this difference is zero (which is marked by a dashed blue line). The six outliers removed from Figures S2 and S3 (with means smaller than 0.9 and variances smaller than 1.3) were also removed before constructing the box plots.
(TIFF)

Table S1 **Distribution of gender in the study groups.**
(DOC)

Table S2 **Distribution of age at diagnosis (cases) or examination (controls) in the study groups.**
(DOC)

Table S3 **Frequency of non-smokers, former smokers and current smokers in the study groups.**
(DOC)

Table S4 **Chromosomal and data base information on the six analyzed SNPs.**
(DOC)

Table S5 **Testing for Hardy-Weinberg equilibrium.**
(DOC)

Table S6 **Maximum odds ratios of the seven polymorphisms in the subgroups.**
(DOCX)

Table S7 **Stability of the ranks of the top ten individual effects in the total study group.**
(DOC)

Table S8 **Stability of the ranks of the top ten individual effects in the ever smoker group.**
(DOC)

Table S9 **Stability of the ranks of the top ten individual effects in the current smoker group.**
(DOC)

Table S10 **Stability of the ranks of the top ten individual effects in the former smoker group.**
(DOC)

Table S11 **Stability of the ranks of the top ten individual effects in the non-smoker group.**
(DOC)

Table S12 **Stability of the ranks of the top ten two-way interactions in the total study group.**
(DOC)

Table S13 **Stability of the ranks of the top ten two-way interactions in the ever smoker group.**
(DOC)

Table S14 **Stability of the ranks of the top ten two-way interactions in the current smoker group.**
(DOC)

Table S15 **Stability of the ranks of the top ten two-way interactions in the former smoker group.**
(DOC)

Table S16 **Stability of the ranks of the top ten two-way interactions in the non-smoker group.**
(DOC)

Table S17 **Stability of the ranks of the top ten three-way interactions in the total study group.**
(DOC)

Table S18 **Stability of the ranks of the top ten three-way interactions in the ever smoker group.**
(DOC)

Table S19 **Stability of the ranks of the top ten three-way interactions in the current smoker group.**
(DOC)

Table S20 **Stability of the ranks of the top ten three-way interactions in the former smoker group.**
(DOC)

Table S21 **Stability of the ranks of the top ten three-way interactions in the non-smoker group.**
(DOC)

Appendix S1 **Details on the polymorphisms.**
(DOCX)

Appendix S2 **Details on the statistical analysis.**
(DOCX)
