# Correction: Distinct SNP Combinations Confer Susceptibility to Urinary Bladder Cancer in Smokers and Non-Smokers

**DOI:** 10.1371/journal.pone.0137937

**Published:** 2015-09-03

**Authors:** Holger Schwender, Silvia Selinski, Meinolf Blaszkewicz, Rosemarie Marchan, Katja Ickstadt, Klaus Golka, Jan G. Hengstler

The allele name rs1014971 [C] appears incorrectly throughout the paper. The correct allele name should be rs1014971 [A], the major allele in Europeans. Correspondingly, rs1014971 [T] should be rs1014971 [G], the minor allele.

The allele name rs8102137 [C] appears incorrectly throughout the paper. The correct allele name should be rs8102137 [T], the major allele. Correspondingly, rs8102137 [T] should be rs8102137 [C], the minor allele.
