# Supplementary figures and images for: Distinct SNP Combinations Confer Susceptibility to Urinary Bladder Cancer in Smokers and Non-Smokers
Source: PLoS One. 2012 Dec 20;7(12):e51880. doi: 10.1371/journal.pone.0051880 (PMC3527453; doi:10.1371/journal.pone.0051880)

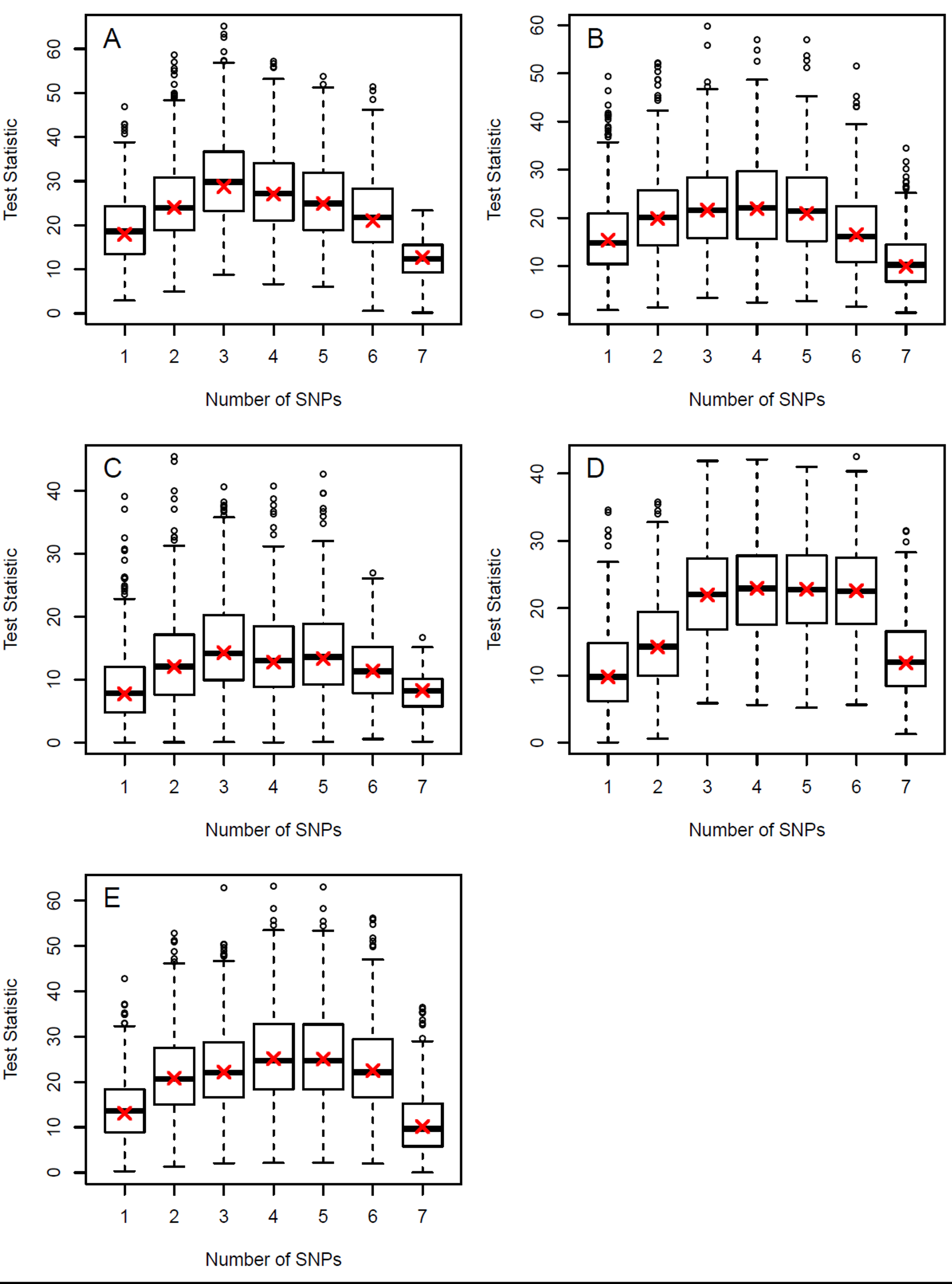

Supplement: Figure S1 — Test statistics for the optimal combinations consisting of one to seven SNPs in the bootstrap samples. Data are shown for (A) the total group, (B) the ever smokers, (C) the current smokers, (D) the former smokers, and (E) the non-smokers. For each of the optimal combinations from in Figure 1A, box plots of the test statistics in the 500 bootstrap samples drawn from the respective subgroups are displayed. The plots correspond to the odds ratios shown in Figure 1B–F. The crosses mark the test statistics for the respective optimal combinations. (TIFF) [file pone.0051880.s001.tiff]

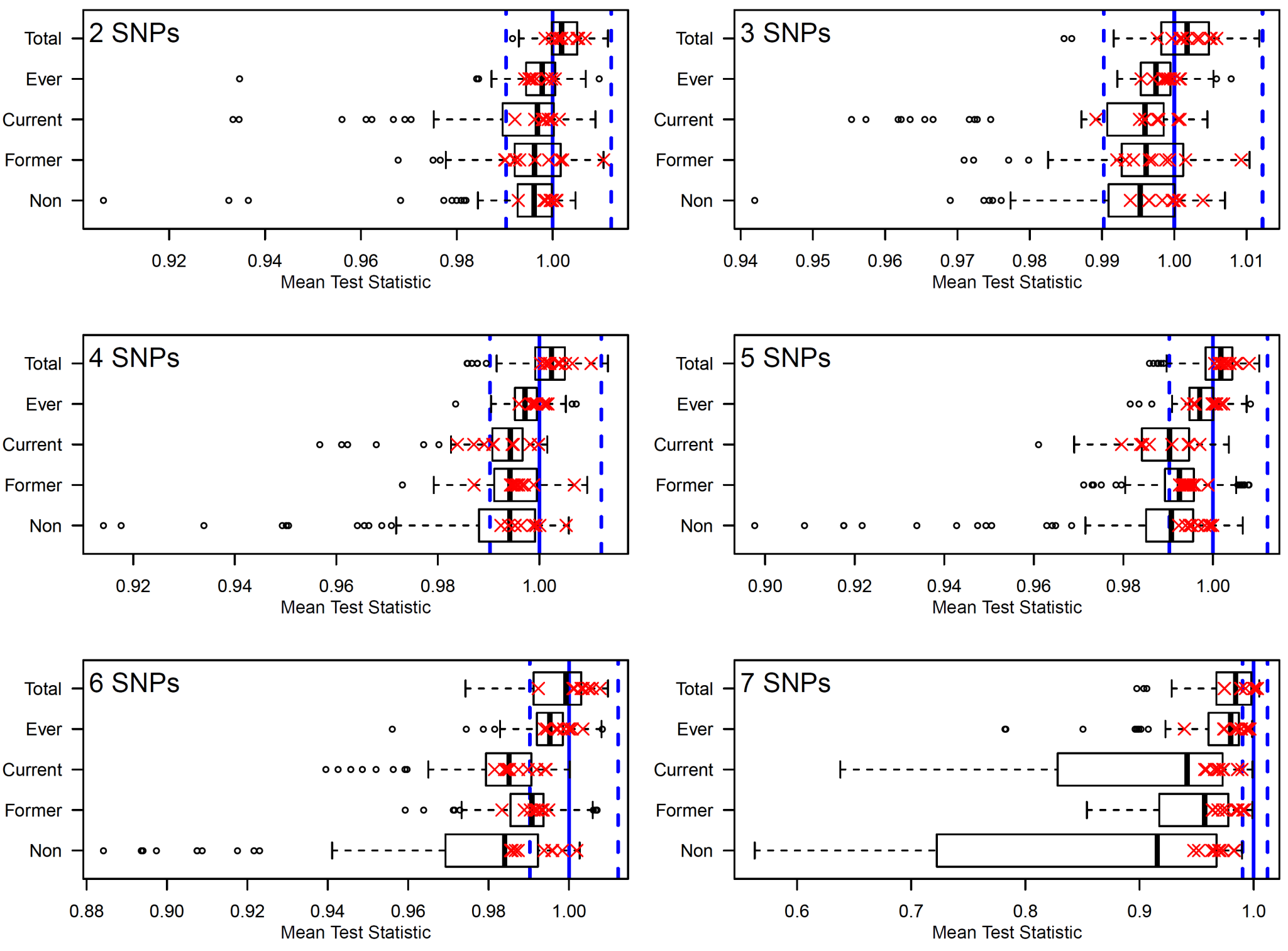

Supplement: Figure S2 — Mean test statistic over 100,000 permutations of the case-control status. For the top 100 SNP combinations of each size and in each subgroup, the means over the Wald statistics in 100,000 permutations of the case-control status were computed. The subgroup-wise distributions of these means are shown as box plots, and the subgroup-wise means of the top 10 combinations are marked by red crosses. For a better representation, six outliers (with means smaller than 0.9) were removed from the box plots for the two-way interactions. For reference, the minimum and maximum of the sample means from 100 samples consisting of 100,000 random draws from a χ2-distribution with 1 degree of freedom are marked by dashed blue lines. If the χ2-approximation is reasonable, the mean test statistic over the 100,000 permutations should be approximately 1, i.e. close to the solid blue lines marking the mean of the χ2-distribution with 1 degree of freedom. (TIFF) [file pone.0051880.s002.tiff]

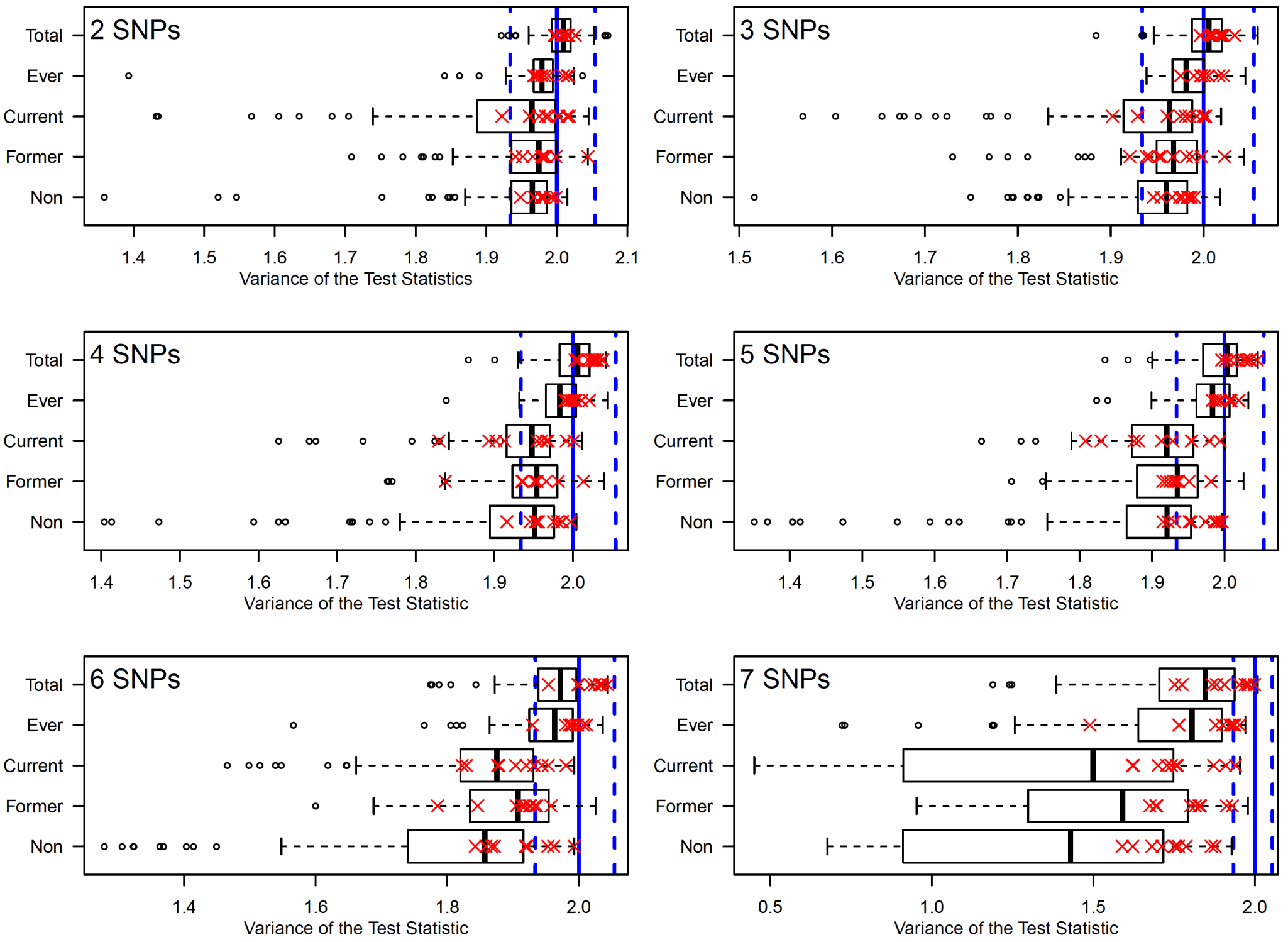

Supplement: Figure S3 — Variance of the test statistic over 100,000 permutations of the case-control status. In addition to the mean test statistic displayed in Figure S2, the variances of the test statistics for the top 100 interactions in the different subgroups were computed. The subgroup-wise distributions of these variances are shown as box plots, and the variances of the top ten combinations in the subgroups are marked by red crosses. For a better representation, six outliers (with variances smaller than 1.3) were removed from the box plots for the two-way interactions. For reference, the minimum and maximum of the sample variances from 100 samples consisting of 100,000 random draws from a χ2-distribution with 1 degree of freedom are marked by dashed blue lines. If the χ2-approximation is reasonable, the variance of the test statistic over the 100,000 permutations should be approximately 2, i.e. close to the solid blue lines marking the variance of the χ2-distribution with 1 degree of freedom. (TIFF) [file pone.0051880.s003.tiff]

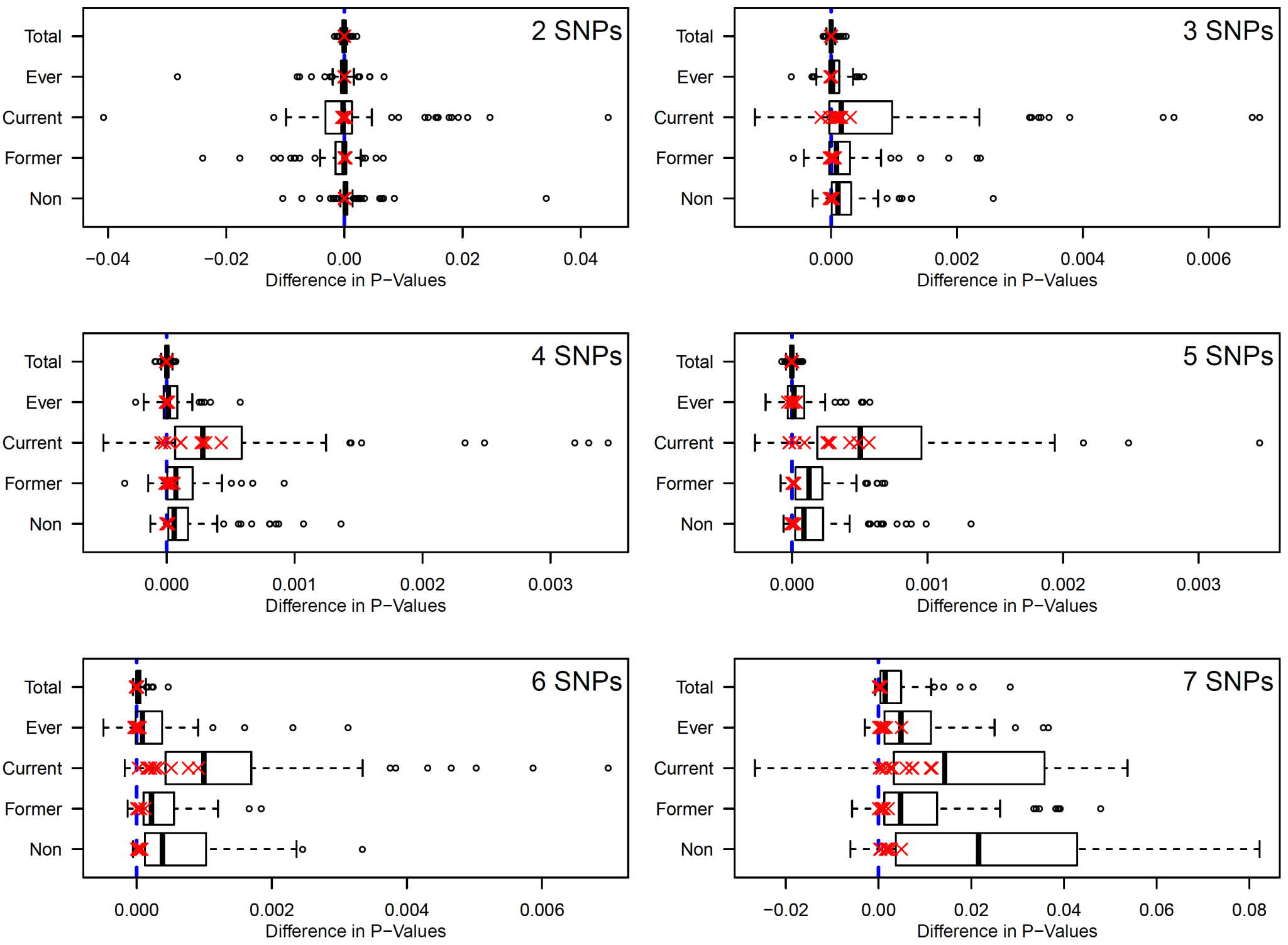

Supplement: Figure S4 — Differences between parametric and permutation-based p-values. Box plots of the differences between the parametric p-values of the top 100 SNP combinations of each size from the analysis of each subgroup and the corresponding p-values computed based on 100,000 permutations of the case-control status. The differences of the respective top ten SNPs are additionally marked by red crosses. Ideally, this difference is zero (which is marked by a dashed blue line). The six outliers removed from Figures S2 and S3 (with means smaller than 0.9 and variances smaller than 1.3) were also removed before constructing the box plots. (TIFF) [file pone.0051880.s004.tiff]
