# Supplementary material for: Distinct SNP Combinations Confer Susceptibility to Urinary Bladder Cancer in Smokers and Non-Smokers
Source: PLoS One. 2012 Dec 20;7(12):e51880. doi: 10.1371/journal.pone.0051880 (PMC3527453; doi:10.1371/journal.pone.0051880)
Supplement: Table S1 — Distribution of gender in the study groups. (DOC) [file pone.0051880.s005.doc]

**Table S1.** Distribution of gender in the study groups.

|  | **Cases** | | **Controls** | |
| --- | --- | --- | --- | --- |
| **Study Group** | **N** | **% Males** | **N** | **% Males** |
| All | 1,595 | 80% | 1,760 | 62% |
| Hungary | 246 | 61% | 78 | 73% |
| Germans Combined | 1,349 | 84% | 1,682 | 61% |
| East Germany | 218 | 86% | 213 | 84% |
| West Germany Combined | 1,131 | 83% | 1,469 | 58% |
| W. Germany – Ongoing | 646 | 77% | 525 | 70% |
| W. Germany – Industrial | 485 | 92% | 944 | 51% |

All consists of the Hungarians and Germans Combined, where Germans Combined is composed of the East Germany Study Group and West Germany Combined, which in turn consists of W. Germany – Ongoing and W. Germany – Industrial.
