# Supplementary material for: Distinct SNP Combinations Confer Susceptibility to Urinary Bladder Cancer in Smokers and Non-Smokers
Source: PLoS One. 2012 Dec 20;7(12):e51880. doi: 10.1371/journal.pone.0051880 (PMC3527453; doi:10.1371/journal.pone.0051880)
Supplement: Table S2 — Distribution of age at diagnosis (cases) or examination (controls) in the study groups. (DOC) [file pone.0051880.s006.doc]

**Table S2.** Distribution of age at diagnosis (cases) or examination (controls) in the study groups.

| **Study Group** | **Status** | **N** | **Mean** | **S.D.** | **Min** | **Median** | **Max** | **N Missing** |
| --- | --- | --- | --- | --- | --- | --- | --- | --- |
| All | Cases | 1,589 | 66.71 | 11.37 | 20.10 | 67.70 | 95.20 | 6 |
|  | Controls | 1,759 | 63.33 | 15.32 | 20.10 | 66.90 | 100.00 | 1 |
| Hungary | Cases | 243 | 69.06 | 11.21 | 27.20 | 70.50 | 95.10 | 3 |
|  | Controls | 77 | 63.52 | 12.69 | 30.00 | 63.40 | 84.20 | 1 |
| Germany Combined | Cases | 1,346 | 66.29 | 11.35 | 20.10 | 67.25 | 95.20 | 3 |
|  | Controls | 1,682 | 63.32 | 15.44 | 20.10 | 66.95 | 100.00 | 0 |
| East Germany | Cases | 218 | 65.04 | 11.28 | 20.10 | 65.70 | 91.30 | 0 |
|  | Controls | 213 | 67.78 | 10.14 | 29.40 | 67.80 | 91.20 | 0 |
| West Germany Combined | Cases | 1,128 | 66.53 | 11.35 | 26.10 | 67.60 | 95.20 | 3 |
|  | Controls | 1,469 | 62.68 | 15.96 | 20.10 | 66.90 | 100.00 | 0 |
| W. Germany – Ongoing | Cases | 646 | 69.82 | 10.90 | 26.10 | 71.30 | 95.20 | 0 |
|  | Controls | 525 | 59.16 | 18.66 | 20.50 | 61.60 | 100.00 | 0 |
| W. Germany – Industrial | Cases | 482 | 62.13 | 10.42 | 32.10 | 62.40 | 84.40 | 3 |
|  | Controls | 944 | 64.63 | 13.87 | 20.10 | 67.75 | 93.90 | 0 |

N: Number of Subjects; S.D.: Standard Deviation; Min: Minimum; Max: Maximum
