# Supplementary material for: Distinct SNP Combinations Confer Susceptibility to Urinary Bladder Cancer in Smokers and Non-Smokers
Source: PLoS One. 2012 Dec 20;7(12):e51880. doi: 10.1371/journal.pone.0051880 (PMC3527453; doi:10.1371/journal.pone.0051880)
Supplement: Table S3 — Frequency of non-smokers, former smokers and current smokers in the study groups. (DOC) [file pone.0051880.s007.doc]

**Table S3.** Frequency of non-smokers, former smokers and current smokers in the study groups.

| **Study Groups** | **Status** | **N** | **Missing** | **Never** | **Former** | **Current** | **Ever** |
| --- | --- | --- | --- | --- | --- | --- | --- |
| All | Cases | 1,494 | 101 | 21% | 50% | 29% | 79% |
|  | Controls | 1,723 | 37 | 44% | 38% | 18% | 56% |
| Hungary | Cases | 226 | 20 | 27% | 36% | 37% | 73% |
|  | Controls | 76 | 2 | 46% | 33% | 21% | 54% |
| Germany Combined | Cases | 1,268 | 81 | 21% | 52% | 27% | 79% |
|  | Controls | 1,647 | 35 | 44% | 38% | 18% | 56% |
| East Germany | Cases | 217 | 1 | 23% | 48% | 29% | 77% |
|  | Controls | 200 | 13 | 47% | 44% | 10% | 54% |
| West Germany Combined | Cases | 1,051 | 80 | 20% | 53% | 27% | 80% |
|  | Controls | 1,447 | 22 | 43% | 38% | 19% | 57% |
| W. Germany – Ongoing | Cases | 624 | 22 | 19% | 57% | 24% | 81% |
|  | Controls | 521 | 4 | 38% | 38% | 24% | 62% |
| W. Germany – Industrial | Cases | 427 | 58 | 21% | 47% | 33% | 79% |
|  | Controls | 926 | 18 | 46% | 37% | 17% | 54% |
