# Supplementary material for: Distinct SNP Combinations Confer Susceptibility to Urinary Bladder Cancer in Smokers and Non-Smokers
Source: PLoS One. 2012 Dec 20;7(12):e51880. doi: 10.1371/journal.pone.0051880 (PMC3527453; doi:10.1371/journal.pone.0051880)
Supplement: Table S4 — Chromosomal and data base information on the six analyzed SNPs. (DOC) [file pone.0051880.s008.doc]

**Table S4. Chromosomal and data base information on the six analyzed SNPs.**

| **SNP** | **Chromosome** | **Position** | **NCBI Build** | **Sequence** |
| --- | --- | --- | --- | --- |
| rs9642880 | 8q24.21 | 128,787,250 | 36.3 | GCAAAGGCTGGAGTTAGGAGAACCC**[G/T]**TGGTTGATGGCTTTGTTAATTATTA |
| rs710521 | 3q28 | 191,128,627 | 36.3 | TTATCAGTGCTTATTCATTTTTTAAG**[A/G]**GATTGAATGCACTAAGGCAAAAGTG |
| rs8102137 | 19q12 | 30,296,853 | 37.3 | GCAGGACTACTAGGAGGCTCACTGG**[C/T]**AGGCACAAATGGGCCTGCCCAGGAC |
| rs11892031 | 2q37.1 | 234,565,283 | 37.3 | TCTGATTGCCACAAAAAGTCCACTG**[A/C]**GTCCATCTCATCTCTATTTTAACAC |
| rs1014971 | 22q13.1 | 39,332,623 | 37.3 | GACGCTGACTCACTGCTGCAGGGGT**[C/T]**GGGAGTACCTGGGTCTCAGCTCTGC |
| rs1495741 | 8p22 | 18,272,881 | 37.3 | GCTGAAGGATGATTTTCATAATAAT**[A/G]** TGGGCATTCACAGTAGCTTCAGGGC |
