# Supplementary material for: Distinct SNP Combinations Confer Susceptibility to Urinary Bladder Cancer in Smokers and Non-Smokers
Source: PLoS One. 2012 Dec 20;7(12):e51880. doi: 10.1371/journal.pone.0051880 (PMC3527453; doi:10.1371/journal.pone.0051880)
Supplement: Table S5 — Testing for Hardy-Weinberg equilibrium. (DOC) [file pone.0051880.s009.doc]

**Table S5. Testing for Hardy-Weinberg equilibrium.**

| **SNP** | **Status** | **N** | **Genotype** | | | | | | **P-value** |
| --- | --- | --- | --- | --- | --- | --- | --- | --- | --- |
| rs9642880 |  |  | G/G | | G/T | | T/T | |  |
|  | Cases | 1,584 | 391 | 25% | 767 | 48% | 426 | 27% | 0.230 |
|  | Controls | 1,738 | 486 | 28% | 876 | 50% | 376 | 22% | 0.639 |
| rs710521 |  |  | A/A | | A/T | | T/T | |  |
|  | Cases | 1,551 | 884 | 57% | 568 | 37% | 99 | 6% | 0.583 |
|  | Controls | 1722 | 907 | 53% | 693 | 40% | 122 | 7% | 0.538 |
| rs1014971 |  |  | C/C | | C/T | | T/T | |  |
|  | Cases | 1,588 | 707 | 45% | 700 | 44% | 181 | 11% | 0.730 |
|  | Controls | 1,719 | 721 | 42% | 799 | 46% | 199 | 12% | 0.339 |
| rs8102137 |  |  | C/C | | C/T | | T/T | |  |
|  | Cases | 1,583 | 641 | 40% | 735 | 46% | 207 | 13% | 0.903 |
|  | Controls | 1,717 | 752 | 44% | 768 | 45% | 197 | 11% | 0.989 |
| rs11892031 |  |  | A/A | | A/C | | C/C | |  |
|  | Cases | 1,592 | 1405 | 88% | 182 | 11% | 5 | 0% | 0.885 |
|  | Controls | 1,725 | 1485 | 86% | 230 | 13% | 10 | 1% | 0.863 |
| rs1495741 |  |  | A/A | | A/G | | G/G | |  |
|  | Cases | 1,588 | 979 | 62% | 541 | 34% | 68 | 4% | 0.576 |
|  | Controls | 1,723 | 1027 | 60% | 623 | 36% | 73 | 4% | 0.085 |
| *GSTM1* |  |  | Present | | Null | |  |  |  |
|  | Cases | 1,572 | 663 | 42% | 909 | 58% |  |  |  |
|  | Controls | 1,739 | 876 | 50% | 863 | 50% |  |  |  |

The SNPs rs9642880, rs710521, rs1014971, rs8102137, rs11892031 and rs1495741 are in Hardy-Weinberg equilibrium (HWE) in all study groups and *GSTM1* shows a distribution typically for Caucasian populations (controls) and UBC cases. N denotes the number of cases or controls, respectively, with non-missing genotypes.
