# Supplementary material for: Distinct SNP Combinations Confer Susceptibility to Urinary Bladder Cancer in Smokers and Non-Smokers
Source: PLoS One. 2012 Dec 20;7(12):e51880. doi: 10.1371/journal.pone.0051880 (PMC3527453; doi:10.1371/journal.pone.0051880)
Supplement: Table S6 — Maximum odds ratios of the seven polymorphisms in the subgroups. (DOCX) [file pone.0051880.s010.docx]

**Table S6.** Maximum odds ratios of the seven polymorphisms in the subgroups.

|  | **Odds Ratio, 95% Confidence Interval and Form of Polymorphism** | | | | |
| --- | --- | --- | --- | --- | --- |
| **Polymorphism^a^** | **All** | **Ever** | **Current** | **Former** | **Non** |
| *GSTM1* [2] | 1.35* | 1.41* | 1.52* | 1.35 | 1.20 |
|  | (1.17-1.56) | (1.19-1.68) | (1.13-2.04) | (1.09-1.67) | (0.92-1.56) |
|  | null | null | null | null | null |
| rs9642880 [7] | 1.34 | 1.33 | 0.87 | 1.50* | 1.48 |
|  | (1.14-1.58) | (1.09-1.63) | (0.62-1.21) | (1.16-1.93) | (1.10-1.99) |
|  | [T/T] | [T/T] | [G/T, T/T] | [T/T] | [T/T] |
| rs710521 [7] | 0.84 | 0.88 | 1.65 | 0.85 | 0.79 |
|  | (0.73-0.97) | (0.74-1.05) | (0.86-3.17) | (0.68-1.05) | (0.60-1.03) |
|  | [A/G, G/G] | [A/G, G/G] | [G/G] | [A/G, G/G] | [A/G, G/G] |
| rs8102137 [9] | 1.18 | 1.14 | 1.19 | 1.12 | 1.51 |
|  | (1.02-1.36) | (0.96-1.36) | (0.88-1.60) | (0.91-1.39) | (1.02-2.24) |
|  | [C/T, T/T] | [C/T, T/T] | [C/T, T/T] | [C/T, T/T] | [T/T] |
| rs11892031 [9] | 0.80 | 0.79 | 0.60 | 0.91 | 0.86 |
|  | (0.65-0.98) | (0.61-1.02) | (0.38-0.93) | (0.66-1.25) | (0.58-1.27) |
|  | [C/C] | [A/C, C/C] | [A/C, C/C] | [A/C, C/C] | [A/C, C/C] |
| rs1014971 [9] | 0.89 | 1.04 | 1.17 | 1.05 | 0.61* |
|  | (0.78-1.03) | (0.87-1.23) | (0.87-1.57) | (0.74-1.50) | (0.47-0.80) |
|  | [C/T, T/T] | [C/T, T/T] | [C/T, T/T] | [T/T] | [C/T, T/T] |
| rs1495741 [9,20] | 0.91 | 0.91 | 0.87 | 0.93 | 0.92 |
|  | (0.79-1.05) | (0.60-1.38) | (0.43-1.75) | (0.55-1.56) | (0.71-1.20) |
|  | [A/G, G/G] | [G/G] | [G/G] | [G/G] | [A/G, G/G] |

Considered are the six SNPs identified in previous genome-wide association studies as well as *GSTM1*. For the SNPs, the maximum odds ratios in the total study group and the different smoker subgroups are determined considering a dominant and a recessive effect of the risk allele. The polymorphism with the largest p-value in a subgroup is marked by an asterisk. Genotypes at risk are given in parenthesis.

^a^ The numbers in the brackets refer to the (genome-wide) association study in which the respective polymorphisms were found to be associated with urinary bladder cancer and correspond to the references in the main manuscript relating to this supporting information.
