# Supplementary material for: Distinct SNP Combinations Confer Susceptibility to Urinary Bladder Cancer in Smokers and Non-Smokers
Source: PLoS One. 2012 Dec 20;7(12):e51880. doi: 10.1371/journal.pone.0051880 (PMC3527453; doi:10.1371/journal.pone.0051880)
Supplement: Table S7 — Stability of the ranks of the top ten individual effects in the total study group. (DOC) [file pone.0051880.s011.doc]

**Table S7. Stability of the ranks of the top ten individual effects in the total study group.**

|  | **Rank in 500 bootstrap samples** | | | |  |
| --- | --- | --- | --- | --- | --- |
| **SNP coding** | **1-3** | **4-6** | **7-10** | **>10** | **OR (95% CI)** |
| *GSTM1* null | 477 | 21 | 2 | 0 | 1.35 (1.18-1.56) |
| rs9642880 [T/T] | 391 | 94 | 9 | 6 | 1.34 (1.14-1.58) |
| rs710521 [A/G, G/G] | 170 | 201 | 110 | 19 | 0.84 (0.73-0.97) |
| rs8102137 [C/T, T/T] | 115 | 193 | 157 | 35 | 1.18 (1.02-1.36) |
| rs11892031 [A/C, C/C] | 120 | 182 | 147 | 51 | 0.80 (0.65-0.98) |
| rs9642880 [G/T, TT] | 87 | 169 | 172 | 72 | 1.16 (0.99-1.36) |
| rs11892031 [C/C] | 8 | 158 | 224 | 110 | 0.34 (0.09-1.24) |
| rs1014971 [C/T, T/T] | 59 | 134 | 202 | 105 | 0.89 (0.78-1.03) |
| rs1495741 [A/G, G/G] | 34 | 106 | 208 | 152 | 0.91 (0.79-1.05) |
| rs8102137 [T/T] | 14 | 90 | 217 | 179 | 1.14 (0.92-1.41) |

The top ten of the 13 variables, either specifying the *GSTM1* genotype or coding for a dominant or recessive effect of the six SNPs, are listed according to their p-values. The stability of these variables was examined by computing their ranks in 500 bootstrap samples from the original data. Moreover, the odds ratios (OR) and the corresponding 95% confidence intervals (95% CI) of these ten variables in the original analysis are shown.
