# Supplementary material for: Distinct SNP Combinations Confer Susceptibility to Urinary Bladder Cancer in Smokers and Non-Smokers
Source: PLoS One. 2012 Dec 20;7(12):e51880. doi: 10.1371/journal.pone.0051880 (PMC3527453; doi:10.1371/journal.pone.0051880)
Supplement: Table S8 — Stability of the ranks of the top ten individual effects in the ever smoker group. (DOC) [file pone.0051880.s012.doc]

**Table S8. Stability of the ranks of the top ten individual effects in the ever smoker group.**

|  | **Rank in 500 bootstrap samples** | | | |  |
| --- | --- | --- | --- | --- | --- |
| **SNP coding** | **1-3** | **4-6** | **7-10** | **>10** | **OR (95% CI)** |
| *GSTM1* null | 487 | 12 | 1 | 0 | 1.41 (1.19-1.68) |
| rs9642880 [T/T] | 394 | 79 | 22 | 5 | 1.33 (1.09-1.63) |
| rs11892031 [A/C, C/C] | 203 | 155 | 110 | 32 | 0.79 (0.61-1.02) |
| rs8102137 [C/T, T/T] | 137 | 200 | 118 | 45 | 1.14 (0.96-1.36) |
| rs710521 [A/G, G/G] | 121 | 171 | 150 | 58 | 0.88 (0.74-1.05) |
| rs1495741 [G/G] | 23 | 147 | 193 | 137 | 0.91 (0.60-1.38) |
| rs9642880 [G/T, T/T] | 22 | 127 | 210 | 141 | 1.04 (0.86-1.27) |
| rs1014971 [C/T, T/T] | 15 | 123 | 199 | 163 | 1.04 (0.87-1.23) |
| rs710521 [G/G] | 28 | 124 | 205 | 143 | 0.94 (0.66-1.32) |
| rs1495741 [A/G, G/G] | 22 | 114 | 199 | 165 | 0.98 (0,82-1.16) |

The top ten of the 13 variables, either specifying the *GSTM1* genotype or coding for a dominant or recessive effect of the six SNPs, are listed according to their p-values. The stability of these variables was examined by computing their ranks in 500 bootstrap samples from the original data. Moreover, the odds ratios (OR) and the corresponding 95% confidence intervals (95% CI) of these ten variables in the original analysis are shown.
