# Supplementary material for: Distinct SNP Combinations Confer Susceptibility to Urinary Bladder Cancer in Smokers and Non-Smokers
Source: PLoS One. 2012 Dec 20;7(12):e51880. doi: 10.1371/journal.pone.0051880 (PMC3527453; doi:10.1371/journal.pone.0051880)
Supplement: Table S9 — Stability of the ranks of the top ten individual effects in the current smoker group. (DOC) [file pone.0051880.s013.doc]

**Table S9. Stability of the ranks of the top ten individual effects in the current smoker group.**

|  | **Rank in 500 bootstrap samples** | | | |  |
| --- | --- | --- | --- | --- | --- |
| **SNP coding** | **1-3** | **4-6** | **7-10** | **>10** | **OR (95%CI)** |
| *GSTM1* null | 427 | 53 | 17 | 3 | 1.52 (1.13-2.04) |
| rs11892031 [A/C, C/C] | 335 | 110 | 44 | 11 | 0.60 (0.38-0.93) |
| rs710521 [G/G] | 188 | 171 | 101 | 40 | 1.65 (0.86-3.17) |
| rs8102137 [C/T, T/T] | 117 | 171 | 152 | 60 | 1.19 (0.88-1.60) |
| rs1014971 [C/T, T/T] | 110 | 152 | 158 | 80 | 1.17 (0.87-1.57) |
| rs9642880 [G/T, T/T] | 86 | 153 | 168 | 93 | 0.87 (0.62-1.21) |
| rs8102137 [T/T] | 59 | 134 | 207 | 100 | 0.86 (0.56-1.34) |
| rs1495741 [G/G] | 37 | 124 | 222 | 117 | 0.87 (0.43-1.75) |
| rs9642880 [T/T] | 38 | 123 | 221 | 118 | 1.07 (0.76-1.50) |
| rs1495741 [A/G, G/G] | 38 | 103 | 230 | 129 | 0.95 (0.70-1.28) |

The top ten of the 13 variables, either specifying the *GSTM1* genotype or coding for a dominant or recessive effect of the six SNPs, are listed according to their p-values. The stability of these variables was examined by computing their ranks in 500 bootstrap samples from the original data. Moreover, the odds ratios (OR) and the corresponding 95% confidence intervals (95% CI) of these ten variables in the original analysis are shown.
