# Supplementary material for: Distinct SNP Combinations Confer Susceptibility to Urinary Bladder Cancer in Smokers and Non-Smokers
Source: PLoS One. 2012 Dec 20;7(12):e51880. doi: 10.1371/journal.pone.0051880 (PMC3527453; doi:10.1371/journal.pone.0051880)
Supplement: Table S10 — Stability of the ranks of the top ten individual effects in the former smoker group. (DOC) [file pone.0051880.s014.doc]

**Table S10. Stability of the ranks of the top ten individual effects in the former smoker group.**

|  | **Rank in 500 bootstrap samples** | | | |  |
| --- | --- | --- | --- | --- | --- |
| **SNP coding** | **1-3** | **4-6** | **6-10** | **>10** | **OR (95% CI)** |
| rs9642880 [T/T] | 448 | 44 | 8 | 0 | 1.50 (1.16-1.93) |
| *GSTM1* null | 393 | 80 | 24 | 3 | 1.35 (1.09-1.67) |
| rs710521 [A/G, G/G] | 163 | 172 | 117 | 48 | 0.85 (0.68-1.05) |
| rs710521 [G/G] | 144 | 172 | 121 | 63 | 0.74 (0.49-1.12) |
| rs9642880 [G/T, T/T] | 92 | 176 | 157 | 75 | 1.15 (0.90-1.46) |
| rs8102137 [C/T, T/T] | 82 | 175 | 148 | 95 | 1.12 (0.91-1.39) |
| rs11892031 [A/C, C/C] | 43 | 115 | 213 | 129 | 0.91 (0.66-1.25) |
| rs8102137 [T/T] | 28 | 119 | 212 | 141 | 1.07 (0.78-1.47) |
| rs1014971 [T/T] | 27 | 110 | 197 | 166 | 1.05 (0.74-1.50) |
| rs1495741 [G/G] | 25 | 117 | 210 | 148 | 0.93 (0.55-1.56) |

The top ten of the 13 variables, either specifying the *GSTM1* genotype or coding for a dominant or recessive effect of the six SNPs, are listed according to their p-values. The stability of these variables was examined by computing their ranks in 500 bootstrap samples from the original data. Moreover, the odds ratios (OR) and the corresponding 95% confidence intervals (95% CI) of these ten variables in the original analysis are shown.
