# Supplementary material for: Distinct SNP Combinations Confer Susceptibility to Urinary Bladder Cancer in Smokers and Non-Smokers
Source: PLoS One. 2012 Dec 20;7(12):e51880. doi: 10.1371/journal.pone.0051880 (PMC3527453; doi:10.1371/journal.pone.0051880)
Supplement: Table S11 — Stability of the ranks of the top ten individual effects in the non-smoker group. (DOC) [file pone.0051880.s015.doc]

**Table S11. Stability of the ranks of the top ten individual effects in the non-smoker group.**

|  | **Rank in 500 bootstrap samples** | | | |  |
| --- | --- | --- | --- | --- | --- |
| **SNP coding** | **1-3** | **4-6** | **7-10** | **>10** | **OR (95% CI)** |
| rs1014971 [C/T, T/T] | 448 | 42 | 9 | 1 | 0.61 (0.47-0.80) |
| rs9642880 [T/T] | 268 | 158 | 67 | 7 | 1.48 (1.10-1.99) |
| rs9642880 [G/T, T/T] | 256 | 169 | 64 | 11 | 1.47 (1.08-2.01) |
| rs8102137 [T/T] | 159 | 194 | 119 | 28 | 1.51 (1.02-2.24) |
| rs710521 [A/G, G/G] | 107 | 187 | 169 | 37 | 0.79 (0.60-1.03) |
| rs8102137 [C/T, T/T] | 80 | 152 | 195 | 73 | 1.22 (0.93-1.59) |
| *GSTM1* null | 74 | 147 | 184 | 95 | 1.20 (0.92-1.56) |
| rs710521 [G/G] | 44 | 162 | 222 | 72 | 0.68 (0.38-1.20) |
| rs11892031 [A/C, C/C] | 25 | 82 | 238 | 155 | 0.86 (0.58-1.27) |
| rs1495741 [A/G, G/G] | 23 | 92 | 244 | 141 | 0.92 (0.71-1.20) |

The top ten of the 13 variables, either specifying the *GSTM1* genotype or coding for a dominant or recessive effect of the six SNPs, are listed according to their p-values. The stability of these variables was examined by computing their ranks in 500 bootstrap samples from the original data. Moreover, the odds ratios (OR) and the corresponding 95% confidence intervals (95% CI) of these ten variables in the original analysis are shown.
