# Supplementary material for: Distinct SNP Combinations Confer Susceptibility to Urinary Bladder Cancer in Smokers and Non-Smokers
Source: PLoS One. 2012 Dec 20;7(12):e51880. doi: 10.1371/journal.pone.0051880 (PMC3527453; doi:10.1371/journal.pone.0051880)
Supplement: Table S12 — Stability of the ranks of the top ten two-way interactions in the total study group. (DOC) [file pone.0051880.s016.doc]

**Table S12.** Stability of the ranks of the top ten two-way interactions in the total study group.

|  | **Rank in 500 bootstrap samples** | | | |  |
| --- | --- | --- | --- | --- | --- |
| **SNP combination^a^** | **1-10** | **11-20** | **21-50** | **>50** | **OR (95% CI)** |
| rs11892031 [A/A] × *GSTM1* null | 386 | 68 | 43 | 3 | 1.42 (1.23-1.63) |
| rs9642880 [G/G, G/T] × *GSTM1* present | 362 | 89 | 42 | 7 | 0.70 (0.60-0.81) |
| rs710521 [A/A, A/G] × *GSTM1* null | 393 | 63 | 41 | 3 | 1.41 (1.23-1.63) |
| rs8102137 [C/T, T/T] × *GSTM1* null | 285 | 121 | 84 | 10 | 1.43 (1.23-1.66) |
| rs9642880 [G/T, T/T] × *GSTM1* null | 277 | 124 | 90 | 9 | 1.39 (1.21-1.61) |
| rs710521 [A/A, A/G] × *GSTM1* present | 257 | 124 | 99 | 20 | 0.73 (0.63-0.84) |
| rs11892031 [A/A, A/C] × *GSTM1* null | 261 | 122 | 102 | 15 | 1.36 (1.19-1.57) |
| rs9642880 [T/T] x rs710521 [A/A, A/G] | 206 | 125 | 133 | 36 | 1.43 (1.21-1.70) |
| rs710521 [A/A] × *GSTM1* null | 185 | 154 | 125 | 36 | 1.39 (1.19-1.62) |
| rs11892031 [A/A, A/C] × *GSTM1* present | 161 | 166 | 140 | 33 | 0.74 (0.65-0.86) |

The top ten of the 288 possible two-way interactions comprised by the six SNPs and *GSTM1* are listed according to their p-values. The stability of these interactions was examined by computing their ranks in 500 bootstrap samples from the original data. Moreover, the odds ratios (OR) and the corresponding 95% confidence intervals (95% CI) of these ten variables in the original analysis are shown.

**^a^** All (unadjusted) p-values are <0.00004.
