# Supplementary material for: Distinct SNP Combinations Confer Susceptibility to Urinary Bladder Cancer in Smokers and Non-Smokers
Source: PLoS One. 2012 Dec 20;7(12):e51880. doi: 10.1371/journal.pone.0051880 (PMC3527453; doi:10.1371/journal.pone.0051880)
Supplement: Table S13 — Stability of the ranks of the top ten two-way interactions in the ever smoker group. (DOC) [file pone.0051880.s017.doc]

**Table S13.** Stability of the ranks of the top ten two-way interactions in the ever smoker group.

|  | **Rank in 500 bootstrap samples** | | | |  |
| --- | --- | --- | --- | --- | --- |
| **SNP combination^a^** | **1-10** | **11-20** | **21-50** | **>50** | **OR (95% CI)** |
| rs11892031 [A/A] × *GSTM1* null | 420 | 53 | 22 | 5 | 1.48 (1.25-1.76) |
| rs8102137 [C/T, T/T] × *GSTM1* null | 358 | 89 | 48 | 5 | 1.51 (1.25-1.82) |
| rs710521 [A/A, A/G] × *GSTM1* null | 364 | 85 | 43 | 8 | 1.46 (1.22-1.73) |
| rs710521 [A/A, A/G] × *GSTM1* present | 330 | 98 | 58 | 14 | 0.69 (0.58-0.83) |
| rs9642880 [G/G, G/T] × *GSTM1* present | 314 | 120 | 56 | 10 | 0.69 (0.57-0.82) |
| rs11892031 [A/A, A/C] × *GSTM1* present | 321 | 91 | 74 | 14 | 0.70 (0.59-0.84) |
| rs11892031 [A/A, A/C] × *GSTM1* null | 316 | 90 | 77 | 17 | 1.42 (1.19-1.69) |
| rs1014971 [C/C, C/T] × *GSTM1* present | 230 | 163 | 85 | 22 | 0.71 (0.60-0.84) |
| rs1495741 [A/A, A/G] × *GSTM1* null | 228 | 149 | 97 | 26 | 1.40 (1.18-1.66) |
| rs1014971 [C/C, C/T] × *GSTM1* null | 160 | 178 | 127 | 35 | 1.38 (1.16-1.64) |

The top ten of the 288 possible two-way interactions comprised by the six SNPs and *GSTM1* are listed according to their p-values. The stability of these interactions was examined by computing their ranks in 500 bootstrap samples from the original data. Moreover, the odds ratios (OR) and the corresponding 95% confidence intervals (95% CI) of these ten variables in the original analysis are shown.

**^a^** All (unadjusted) p-values are <0.0003.
