# Supplementary material for: Distinct SNP Combinations Confer Susceptibility to Urinary Bladder Cancer in Smokers and Non-Smokers
Source: PLoS One. 2012 Dec 20;7(12):e51880. doi: 10.1371/journal.pone.0051880 (PMC3527453; doi:10.1371/journal.pone.0051880)
Supplement: Table S14 — Stability of the ranks of the top ten two-way interactions in the current smoker group. (DOC) [file pone.0051880.s018.doc]

**Table S14.** Stability of the ranks of the top ten two-way interactions in the current smoker group.

|  | **Rank in 500 bootstrap samples** | | | |  |
| --- | --- | --- | --- | --- | --- |
| **SNP combination^a^** | **1-10** | **11-20** | **21-50** | **>50** | **OR (95% CI)** |
| rs11892031 [A/A] × *GSTM1* null | 357 | 69 | 58 | 16 | 1.69 (1.26-2.27) |
| rs1014971 [C/C, C/T] × *GSTM1* present | 292 | 89 | 83 | 36 | 0.61 (0.45-0.83) |
| rs1014971 [C/C, C/T] × *GSTM1* null | 293 | 88 | 72 | 47 | 1.62 (1.20-2.17) |
| rs9642880 [G/G, G/T] × *GSTM1* present | 202 | 126 | 107 | 65 | 0.63 (0.46-0.85) |
| rs8102137 [C/T, T/T] × *GSTM1* null | 205 | 132 | 97 | 66 | 1.61 (1.17-2.22) |
| rs710521 [A/G, G/G] × *GSTM1* present | 192 | 102 | 116 | 90 | 0.57 (0.39-0.83) |
| rs710521 [A/A, A/G] × *GSTM1* present | 213 | 92 | 129 | 66 | 0.64 (0.48-0.87) |
| rs11892031 [A/A, A/C] × *GSTM1* present | 179 | 122 | 124 | 75 | 0.65 (0.49-0.88) |
| rs11892031 [A/A, A/C] × *GSTM1* null | 149 | 128 | 134 | 89 | 1.47 (1.09-1.98) |
| rs1014971 [C/C] × *GSTM1* present | 154 | 104 | 157 | 85 | 0.59 (0.40-0.86) |

The top ten of the 288 possible two-way interactions comprised by the six SNPs and *GSTM1* are listed according to their p-values. The stability of these interactions was examined by computing their ranks in 500 bootstrap samples from the original data. Moreover, the odds ratios (OR) and the corresponding 95% confidence intervals (95% CI) of these ten variables in the original analysis are shown.

**^a^** All (unadjusted) p-values are <0.007.
