# Supplementary material for: Distinct SNP Combinations Confer Susceptibility to Urinary Bladder Cancer in Smokers and Non-Smokers
Source: PLoS One. 2012 Dec 20;7(12):e51880. doi: 10.1371/journal.pone.0051880 (PMC3527453; doi:10.1371/journal.pone.0051880)
Supplement: Table S15 — Stability of the ranks of the top ten two-way interactions in the former smoker group. (DOC) [file pone.0051880.s019.doc]

**Table S15.** Stability of the ranks of the top ten two-way interactions in the former smoker group.

|  | **Rank in 500 bootstrap samples** | | | |  |
| --- | --- | --- | --- | --- | --- |
| **SNP combinations^a^** | **1-10** | **11-20** | **21-50** | **>50** | **OR (95% CI)** |
| rs9642880 [T/T] × *GSTM1* null | 323 | 96 | 69 | 12 | 1.86 (1.35-2.57) |
| rs710521 [A/A, A/G] × *GSTM1* null | 296 | 91 | 89 | 24 | 1.49 (1.21-1.85) |
| rs9642880 [T/T] × rs8102137 [C/T, T/T] | 287 | 85 | 106 | 22 | 1.83 (1.32-2.55) |
| rs710521 [A/A] × *GSTM1* null | 224 | 102 | 135 | 39 | 1.51 (1.19-1.92) |
| rs9642880 [T/T] × rs1495741 [A/A, A/G] | 226 | 98 | 123 | 53 | 1.54 (1.19-2.00) |
| rs9642880 [T/T] × rs710521 [A/A, A/G] | 195 | 116 | 135 | 54 | 1.54 (1.19-2.00) |
| rs8102137 [C/T, T/T] × *GSTM1* null | 184 | 104 | 140 | 72 | 1.46 (1.16-1.84) |
| rs9642880 [T/T] × rs1014971 [C/T, T/T] | 162 | 99 | 159 | 80 | 1.66 (1.21-2.28) |
| rs962880 [T/T] × rs11892031 [A/A, A/C] | 175 | 98 | 151 | 76 | 1.49 (1.16-1.92) |
| rs962880 [G/G, G/T] × rs11892031 [A/A, A/C] | 158 | 106 | 150 | 86 | 0.68 (0.53-0.87) |

The top ten of the 288 possible two-way interactions comprised by the six SNPs and *GSTM1* are listed according to their p-values. The stability of these interactions was examined by computing their ranks in 500 bootstrap samples from the original data. Moreover, the odds ratios (OR) and the corresponding 95% confidence intervals (95% CI) of these ten variables in the original analysis are shown.

**^a^** All (unadjusted) p-values are <0.003.
