# Supplementary material for: Distinct SNP Combinations Confer Susceptibility to Urinary Bladder Cancer in Smokers and Non-Smokers
Source: PLoS One. 2012 Dec 20;7(12):e51880. doi: 10.1371/journal.pone.0051880 (PMC3527453; doi:10.1371/journal.pone.0051880)
Supplement: Table S16 — Stability of the ranks of the top ten two-way interactions in the non-smoker group. (DOC) [file pone.0051880.s020.doc]

**Table S16.** Stability of the ranks of the top ten two-way interactions in the non-smoker group.

|  | **Rank in 500 bootstrap samples** | | | |  |
| --- | --- | --- | --- | --- | --- |
| **SNP combination^a^** | **1-10** | **11-20** | **21-50** | **>50** | **OR (95% CI)** |
| rs9642880 [G/T, T/T] × rs1014971 [C/C] | 421 | 43 | 23 | 13 | 1.91 (1.44-2.51) |
| rs9642880 [G/G, G/T] × rs1014971 [C/T, T/T] | 331 | 97 | 61 | 11 | 0.56 (0.43-0.74) |
| rs710521 [A/A, A/G] × rs1014971 [C/C] | 262 | 98 | 104 | 36 | 1.68 (1.28-2.20) |
| rs1014971 [C/C] × rs1495741 [A/A, A/G] | 254 | 104 | 102 | 40 | 1.66 (1.27-2.16) |
| rs1014971 [C/C] × rs11892031 [A/A, A/C] | 259 | 94 | 105 | 42 | 1.65 (1.27-2.16) |
| rs1014971 [C/T, T/T] × rs8102137 [C/C, C/T] | 221 | 118 | 114 | 47 | 0.61 (0.46-0.79) |
| rs1014971 [C/C] × rs11892031 [A/A] | 206 | 117 | 121 | 56 | 1.65 (1.26-2.15) |
| rs9642880 [T/T] × rs710521 [A/A, A/G] | 191 | 111 | 146 | 52 | 1.75 (1.29-2.37) |
| rs1014971 [C/T, T/T] × rs1495741 [A/A, A/G] | 185 | 124 | 127 | 64 | 0.62 (0.47-0.81) |
| rs1014971 [C/C] × *GSTM1* null | 171 | 113 | 149 | 67 | 1.73 (1.28-2.35) |

The top ten of the 288 possible two-way interactions comprised by the six SNPs and *GSTM1* are listed according to their p-values. The stability of these interactions was examined by computing their ranks in 500 bootstrap samples from the original data. Moreover, the odds ratios (OR) and the corresponding 95% confidence intervals (95% CI) of these ten variables in the original analysis are shown.

**^a^** All (unadjusted) p-values are <0.0004.
