# Supplementary material for: Distinct SNP Combinations Confer Susceptibility to Urinary Bladder Cancer in Smokers and Non-Smokers
Source: PLoS One. 2012 Dec 20;7(12):e51880. doi: 10.1371/journal.pone.0051880 (PMC3527453; doi:10.1371/journal.pone.0051880)
Supplement: Table S17 — Stability of the ranks of the top ten three-way interactions in the total study group. (DOC) [file pone.0051880.s021.doc]

**Table S17.** Stability of the ranks of the top ten three-way interactions in the total study group.

|  | **Rank in 500 bootstrap samples** | | | |  |
| --- | --- | --- | --- | --- | --- |
| **SNP combination^a^** | **1-10** | **11-20** | **21-50** | **>50** | **OR (95% CI)** |
| rs710521 [A/A, A/G] × rs11892031 [A/A] × *GSTM1* null | 334 | 68 | 70 | 28 | 1.48 (1.28-1.70) |
| rs9642880 [G/G, G/T] × rs710521 [A/A, A/G] × *GSTM1* present | 286 | 85 | 87 | 42 | 0.67 (0.58-0.78) |
| rs8102137 [C/T, T/T] × rs11892031 [A/A] × *GSTM1* null | 196 | 85 | 127 | 92 | 1.49 (1.27-1.74) |
| rs710521 [A/A,A/G] × rs8102137 [C/T, T/T] × *GSTM1* null | 175 | 82 | 134 | 109 | 1.47 (1.26-1.72) |
| rs710521 [A/A, A/G] × rs11892031 [A/A, A/C] × *GSTM1* null | 168 | 117 | 124 | 91 | 1.41 (1.23-1.63) |
| rs9642880 [G/T, T/T] × rs11892031 [A/A] × *GSTM1* null | 140 | 97 | 117 | 146 | 1.42 (1.23-1.65) |
| rs9642880 [G/G, G/T] × rs11892031 [A/A, A/C] × *GSTM1* present | 136 | 102 | 139 | 123 | 0.70 (0.60-0.81) |
| rs9642880 [G/T, T/T] × rs710521 [A/A, A/G] × *GSTM1* null | 146 | 80 | 146 | 128 | 1.42 (1.22-1.64) |
| rs9642880 [G/G, G/T] × rs1495741 [A/A, A/G] × *GSTM1* present | 113 | 100 | 145 | 142 | 0.70 (0.61-0.82) |
| rs8102137 [C/T, TT] × rs11892031 [A/A, A/C] × *GSTM1* null | 93 | 94 | 148 | 165 | 1.43 (1.23-1.66) |

The top ten of the 1,760 possible three-way interactions comprised by the six SNPs and *GSTM1* are listed according to their p-values. The stability of these interactions was examined by computing their ranks in 500 bootstrap samples from the original data. Moreover, the odds ratios (OR) and the corres­ponding 95% confidence intervals (95% CI) of these ten variables in the original analysis are shown.

^a^ All (unadjusted) p-values are <0.00005.
