# Supplementary material for: Distinct SNP Combinations Confer Susceptibility to Urinary Bladder Cancer in Smokers and Non-Smokers
Source: PLoS One. 2012 Dec 20;7(12):e51880. doi: 10.1371/journal.pone.0051880 (PMC3527453; doi:10.1371/journal.pone.0051880)
Supplement: Table S18 — Stability of the ranks of the top ten three-way interactions in the ever smoker group. (DOC) [file pone.0051880.s022.doc]

**Table S18.** Stability of the ranks of the top ten three-way interactions in the ever smoker group.

|  | **Rank in 500 bootstrap samples** | | | |  |
| --- | --- | --- | --- | --- | --- |
| **SNP combination^a^** | **1-10** | **11-20** | **21-50** | **>50** | **OR (95% CI)** |
| rs8102137 [C/T, T/T] × rs11892031 [A/A] × *GSTM1* null | 291 | 77 | 79 | 53 | 1.58 (1.30-1.92) |
| rs710521 [A/A, A/G] × rs11892031 [A/A] × *GSTM1* null | 291 | 95 | 68 | 46 | 1.51 (1.26-1.80) |
| rs710521 [A/A, A/G] × rs8102137 [C/T, T/T] × *GSTM1* null | 247 | 78 | 102 | 73 | 1.55 (1.28-1.88) |
| rs9642880 [G/G, G/T] × rs710521 [A/A, A/G] × *GSTM1* present | 225 | 85 | 121 | 69 | 0.66 (0.55-0.79) |
| rs8102137 [C/T, T/T] × rs1495741 [A/A, A/G] × *GSTM1* null | 207 | 80 | 129 | 84 | 1.52 (1.26-1.84) |
| rs8102137 [C/T, T/T] × rs11892031 [A/A, A/C] × *GSTM1* null | 190 | 94 | 118 | 98 | 1.50 (1.25-1.81) |
| rs1014971 [C/C, C/T] × rs11892031 [A/A] × *GSTM1* null | 170 | 107 | 121 | 102 | 1.46 (1.22-1.74) |
| rs9642880 [G/G, G/T] × rs11892031 [A/A, A/C] × *GSTM1* present | 159 | 88 | 143 | 110 | 0.68 (0.57-0.82) |
| rs710521 [A/A, A/G] × rs11892031 [A/A, A/C] × *GSTM1* null | 131 | 116 | 145 | 108 | 1.45 (1.22-1.72) |
| rs710521 [A/A, A/G] × rs1014971 [C/C, C/T] × *GSTM1* present | 133 | 101 | 142 | 124 | 0.69 (0.57-0.82) |

The top ten of the 1,760 possible three-way interactions comprised by the six SNPs and *GSTM1* are listed according to their p-values. The stability of these interactions was examined by computing their ranks in 500 bootstrap samples from the original data. Moreover, the odds ratios (OR) and the corres­ponding 95% confidence intervals (95% CI) of these ten variables in the original analysis are shown.

^a^ All (unadjusted) p-values are <0.00004.
