# Supplementary material for: Distinct SNP Combinations Confer Susceptibility to Urinary Bladder Cancer in Smokers and Non-Smokers
Source: PLoS One. 2012 Dec 20;7(12):e51880. doi: 10.1371/journal.pone.0051880 (PMC3527453; doi:10.1371/journal.pone.0051880)
Supplement: Table S19 — Stability of the ranks of the top ten three-way interactions in the current smoker group. (DOC) [file pone.0051880.s023.doc]

**Table S19.** Stability of the ranks of the top ten three-way interactions in the current smoker group.

|  | **Rank in 500 bootstrap samples** | | | |  |
| --- | --- | --- | --- | --- | --- |
| **SNP combination^a^** | **1-10** | **11-20** | **21-50** | **>50** | **OR (95% CI)** |
| rs1014971 [C/C, C/T] × rs11892031 [A/A] × *GSTM1* null | 267 | 84 | 76 | 73 | 1.79 (1.32-2.42) |
| rs710521 [A/A, A/G] × rs1014971 [C/C, C/T] × *GSTM1* present | 169 | 98 | 112 | 121 | 0.58 (0.42-0.79) |
| rs9642880 [G/T, T/T] × rs710521 [A/G, G/G] × *GSTM1* present | 151 | 59 | 114 | 176 | 0.47 (0.30-0.72) |
| rs710521 [A/G, G/G] × rs1014971 [C/T, T/T] × *GSTM1* null | 160 | 62 | 101 | 177 | 2.39 (1.43-3.97) |
| rs1014971 [C/C, C/T] × rs11892031 [A/A, A/C] × *GSTM1* present | 114 | 80 | 130 | 176 | 0.60 (0.45-0.82) |
| rs1014971 [C/C, C/T] × rs8102137 [C/T, T/T] × *GSTM1* null | 141 | 65 | 112 | 182 | 1.75 (1.25-2.45) |
| rs9642880 [G/G, G/T] × rs710521 [A/A, A/G] × *GSTM1* present | 113 | 74 | 132 | 181 | 0.59 (0.43-0.81) |
| rs8102137 [C/C, C/T] × rs11892031 [A/A] × *GSTM1* null | 91 | 63 | 144 | 202 | 1.64 (1.21-2.21) |
| rs1014971 [C/C, C/T] × rs11892031 [A/A, A/C] × *GSTM1* null | 96 | 87 | 131 | 186 | 1.62 (1.20-2.17) |
| rs9642880 [G/G, G/T] × rs1014971 [C/C, C/T] × *GSTM1* present | 102 | 66 | 124 | 208 | 0.60 (0.43-0.82) |

The top ten of the 1,760 possible three-way interactions comprised by the six SNPs and *GSTM1* are listed according to their p-values. The stability of these interactions was examined by computing their ranks in 500 bootstrap samples from the original data. Moreover, the odds ratios (OR) and the corres­ponding 95% confidence intervals (95% CI) of these ten variables in the original analysis are shown.

^a^ All (unadjusted) p-values are <0.002.
