# Supplementary material for: Distinct SNP Combinations Confer Susceptibility to Urinary Bladder Cancer in Smokers and Non-Smokers
Source: PLoS One. 2012 Dec 20;7(12):e51880. doi: 10.1371/journal.pone.0051880 (PMC3527453; doi:10.1371/journal.pone.0051880)
Supplement: Table S20 — Stability of the ranks of the top ten three-way interactions in the former smoker group. (DOC) [file pone.0051880.s024.doc]

**Table S20.** Stability of the ranks of the top ten three-way interactions in the former smoker group.

|  | **Rank in 500 bootstrap samples** | | | |  |
| --- | --- | --- | --- | --- | --- |
| **SNP combination^a^** | **1-10** | **11-20** | **21-50** | **>50** | **OR (95% CI)** |
| rs9642880 [T/T] × rs8102137 [C/T, T/T] × *GSTM1* null | 376 | 60 | 45 | 19 | 2.92 (1.87-4.57) |
| rs9642880 [T/T] × rs710521 [A/A, A/G] × *GSTM1* null | 199 | 87 | 108 | 106 | 1.99 (1.42-2.79) |
| rs9642880 [T/T] × rs8102137 [C/T, T/T] × rs1495741 [A/A, A/G] | 162 | 83 | 114 | 141 | 1.94 (1.39-2.71) |
| rs9642880 [T/T] × rs710521 [A/A] × *GSTM1* null | 125 | 83 | 148 | 144 | 2.37 (1.52-3.70) |
| rs710521 [A/A, A/G] × rs8102137 [C/T, T/T] × *GSTM1* null | 150 | 62 | 120 | 168 | 1.58 (1.24-2.00) |
| rs9642880 [T/T] × rs11892031 [A/A, A/C] × *GSTM1* null | 126 | 97 | 110 | 167 | 1.85 (1.34-2.56) |
| rs9642880 [T/T] × rs710521 [A/A, A/G] × rs8102137 [C/T, T/T] | 123 | 75 | 133 | 169 | 1.91 (1.36-2.70) |
| rs9642880 [T/T] × rs8102137 [C/T, T/T] × rs11892031 [A/A] | 123 | 77 | 125 | 176 | 1.91 (1.35-2.70) |
| rs9642880 [T/T] × rs1495741 [A/A, A/G] × *GSTM1* null | 103 | 76 | 137 | 184 | 1.84 (1.32-2.56) |
| rs710521 [A/A, A/G] × rs11892031 [A/A, A/C] × *GSTM1* null | 98 | 88 | 120 | 194 | 1.49 (1.20-1.84) |

The top ten of the 1,760 possible three-way interactions comprised by the six SNPs and *GSTM1* are listed according to their p-values. The stability of these interactions was examined by computing their ranks in 500 bootstrap samples from the original data. Moreover, the odds ratios (OR) and the corres­ponding 95% confidence intervals (95% CI) of these ten variables in the original analysis are shown.

^a^ All (unadjusted) p-values are <0.0003.
