# Supplementary material for: Distinct SNP Combinations Confer Susceptibility to Urinary Bladder Cancer in Smokers and Non-Smokers
Source: PLoS One. 2012 Dec 20;7(12):e51880. doi: 10.1371/journal.pone.0051880 (PMC3527453; doi:10.1371/journal.pone.0051880)
Supplement: Table S21 — Stability of the ranks of the top ten three-way interactions in the non-smoker group. (DOC) [file pone.0051880.s025.doc]

**Table S21.** Stability of the ranks of the top ten three-way interactions in the non-smoker group.

|  | **Rank in 500 bootstrap samples** | | | |  |
| --- | --- | --- | --- | --- | --- |
| **SNP combination^a^** | **1-10** | **11-20** | **21-50** | **>50** | **OR (95% CI)** |
| rs9642880 [G/T, T/T] × rs710521 [A/A, A/G] × rs1014971 [C/C] | 300 | 78 | 74 | 48 | 1.98 (1.49-2.63) |
| rs9642880 [G/T, T/T] × rs1014971 [C/C] × rs1495741 [A/A, A/G] | 291 | 75 | 83 | 51 | 1.95 (1.47-2.58) |
| rs9642880 [G/T, T/T] × rs1014971 [C/C] × rs11892031 [A/A, A/C] | 283 | 82 | 80 | 55 | 1.93 (1.46-2.55) |
| rs9642880 [G/T, T/T] × rs1014971 [C/C] × *GSTM1* null | 246 | 82 | 95 | 77 | 2.21 (1.58-3.10) |
| rs9642880 [G/G, G/T] × rs1014971 [C/T, T/T] × rs8102137 [C/C, C/T] | 173 | 100 | 134 | 93 | 0.54 (0.40-0.71) |
| rs9642880 [G/G, G/T] × rs710521 [A/A, A/G] × rs1014971 [C/T, T/T] | 166 | 114 | 125 | 95 | 0.54 (0.40-0.71) |
| rs9642880 [G/G, G/T] × rs1014971 [C/T, T/T] × rs1495741 [A/A, A/G] | 122 | 96 | 149 | 133 | 0.56 (0.42-0.74) |
| rs710521 [A/A, A/G] × rs1014971 [C/C] × *GSTM1* null | 141 | 60 | 134 | 165 | 1.93 (1.41-2.64) |
| rs9642880 [G/T, T/T] × rs1014971 [C/C] × rs11892031 [A/A] | 106 | 92 | 138 | 164 | 1.80 (1.35-2.40) |
| rs710521 [A/A, A/G] × rs1014971 [C/C] × rs11892031 [A/A] | 114 | 84 | 135 | 167 | 1.74 (1.33-2.29) |

The top ten of the 1,760 possible three-way interactions comprised by the six SNPs and *GSTM1* are listed according to their p-values. The stability of these interactions was examined by computing their ranks in 500 bootstrap samples from the original data. Moreover, the odds ratios (OR) and the corres­ponding 95% confidence intervals (95% CI) of these ten variables in the original analysis are shown.

^a^ All (unadjusted) p-values are <0.00007.
