# Supplementary material for: Distinct SNP Combinations Confer Susceptibility to Urinary Bladder Cancer in Smokers and Non-Smokers
Source: PLoS One. 2012 Dec 20;7(12):e51880. doi: 10.1371/journal.pone.0051880 (PMC3527453; doi:10.1371/journal.pone.0051880)
Supplement: Appendix S1 — Details on the polymorphisms. (DOCX) [file pone.0051880.s026.docx]

**Appendix S1. Details on the polymorphisms.**

Blood samples were taken by physicians in the respective hospitals, frozen and transported to our lab, where all further sample processing and SNP analyses were performed. Starting with 2 to 8 mL whole blood, the isolation of leukocyte-DNA was carried out according to the QIAamp^®^ DNA Blood Midi/Maxi Handbook (www.qiagen.com). This method is based on the selective adsorption of nucleic acids to a silica-gel membrane. The yielded DNA amount was diluted with H_2_O to 5 ng/µL for each sample. In order to perform the SNP genotyping studies, custom TaqMan^®^ reagent-based assays from Applied Biosystems, Life Technologies, Germany were used (rs1014971 (assay ID C___2189441_1_), rs11892031 (C__32051717_10), rs1495741 (C___8684110_10), rs710521 (C___8239360_10), rs8102137 (C__29353673_10) and rs9642880 (C__29543302_10). These assays contain a primer pair for the PCR of the specific DNA region and two allele-specific probes with either VIC^®^ or 6-FAM^™^ fluorescent reporter dyes and the quencher group. The complete probe does not emit fluorescence light because of the fluorescence resonance energy transfer (FRET) between the fluorophore and the quencher. Degradation of the probe by the 5'-3'-exonuclease activity of the Taq polymerase during amplification liberates the fluorophore and causes an increase in the fluorescence signal after each PCR cycle, which is detected by the real-time PCR machine (ABI PRISM^®^ 7900HT, Applied Biosystems Life Technologies, Germany). Plotting the fluorescence intensities of VIC^®^ and 6-FAM^™^ for all samples in a 96 well plate resulted in three clusters for the three allelic combinations where the genotypes can be assigned. Starting with 10 ng DNA per well, the threshold cycle appears after 25 – 28 repetitions. Samples with no templates served as controls (NTC) for the fluorescence background. The whole assay is stopped after 40 cycles. To solve the problem of control samples, the following procedure was applied for all six SNPs of this study. The first 92 samples (4 NTCs in a 96 well plate) were analysed without a control. Screening the results, two samples for each allelic combination (homozygous wild/wild, heterozygous wild/mutant and homozygous mutant/mutant) were chosen as controls for all further determinations of this specific SNP. This procedure is very stable and the once determined genotype was confirmed in all following analyses. In this way, the six controls were analysed approximately forty times. The determination of the genotype was done once for each sample, and only questionable results were repeated. Details of the SNPs are given in Table S4. The homozygous *GSTM1* deletion was detected by the amplification of the *GSTM1* DNA sequence segment with 218 base pairs by means of PCR [29,30]. After gel electrophoresis using ethidium bromide, the DNA product was detected using UV light. This method helped determine whether at least one copy of the *GSTM1* gene was present or both copies of the *GSTM1* gene were missing.
