# Supplementary material for: Distinct SNP Combinations Confer Susceptibility to Urinary Bladder Cancer in Smokers and Non-Smokers
Source: PLoS One. 2012 Dec 20;7(12):e51880. doi: 10.1371/journal.pone.0051880 (PMC3527453; doi:10.1371/journal.pone.0051880)
Supplement: Appendix S2 — Details on the statistical analysis. (DOCX) [file pone.0051880.s027.docx]

**Appendix S2. Details on the statistical analysis.**

To determine ORs of the individual SNPs and combinations of these polymorphisms in the total cohort as well as in subgroups defined by the smoking status of the subjects, four binary variables for each of the six SNPs were generated. These variables coded either for a dominant or a recessive mode of inheritance or for the complements of these modes, i.e. for a "protective dominant" or "protective recessive" effect of the SNP. The binary variable for *GSTM1* was set to one if *GSTM1* was negative. In addition, this binary variable, as well as 1 minus this binary variable, were considered in our analysis.

To identify the combination of *p* polymorphisms (*p* = 2, …, 7) showing the OR with the lowest p-value, the ORs for all combinations of the coding variables for *p* of the six SNPs, as well as all combinations of *GSTM1* with the variables coding for *p* – 1 SNPs were computed in each of the subgroups.

The p-values of the Wald tests for investigating whether the ORs differ from one were determined using a χ^2^ distribution with one degree of freedom. To check whether it is also appropriate to compute p-values for higher-order SNP interactions in this parametric way, we also computed p-values based on 100,000 permutations of the case-control status and compared these permutation-based p-values with the parametric p-values. For this comparison, both the means and variances over the test statistics from 100,000 permutations were calculated (where the mean should be approximately 1 and the variance about 2, representing a random variable that asymptotically follows a χ^2^ distribution with one degree of freedom). Additionally, we computed the differences between the parametric and the corresponding permutation-based p-values for the top 100 SNP combinations of each size and in each of the subgroups.

A bootstrap strategy was used to investigate the stability of the ORs of the SNP combinations of different sizes and their respective ranks in the total group as well as in the different smoker subgroups. Thus, we randomly drew *n*_Ca_ cases and *n*_Co_ controls with replacement from the *n*_Ca_ cases and *n*_Co_ controls composing the respective subgroup. The ORs as well as the corresponding Wald statistics were then recomputed, and the ORs were ordered according to their respective p-values. This procedure was repeated 500 times, and the frequencies at which the top 10 SNP combinations from the original analysis appeared among the top 10, top 20, and top 50 SNP combinations (of the same number of SNPs) from the analyses of the corresponding 500 bootstrap samples were recorded.

To test whether the OR of a certain SNP combination differs between the ever smokers and the non-smokers, we fitted the logistic regression model:

logit(Prob(*Y* = 1)) = *β*_0_ + *β*_1_ * *X* + *β*_2_ * *S* + *β*_3_ * (*X* × *S*),

where *X* and *S* are binary variables coding for the SNP combination (i.e *X* = 1, if a subject shows this combination, and *X* = 0, otherwise) and the smoking status (*S* = 0, if a subject is a non-smoker, and *S* = 1, otherwise), respectively, and (*X* × *S*) represents the interaction of the SNP combination with the smoking status. Thus, exp(*β*_1_) is the odds ratio for the SNP combination in the non-smokers and exp(*β*_1_ + *β*_3_) the odds ratio for the SNP combination in the ever smokers. The standard Wald test for the parameter *β*_3_ in the above logistic regression model can therefore be used to test whether the odds ratios differ significantly between smokers and non-smokers.
